# Supplementary material for: Transcriptome analysis during fruit developmental stages in durian (Durio zibethinus Murr.) var. D24
Source: Genet Mol Biol. 2023 Jan 6;45(4):e20210379. doi: 10.1590/1678-4685-GMB-2021-0379 (PMC9830936; doi:10.1590/1678-4685-GMB-2021-0379)
Supplement: Figure S1 - [file 1415-4757-GMB-45-4-e20210379-s1.pdf]

*Genetics and Molecular Biology*
DOI: https://doi.org/10.1590/1678-4685-GMB-2021-0379

# Supplementary Material to "Transcriptome analysis during fruit developmental stages in durian (*Durio zibethinus* Murr.) var. D24"

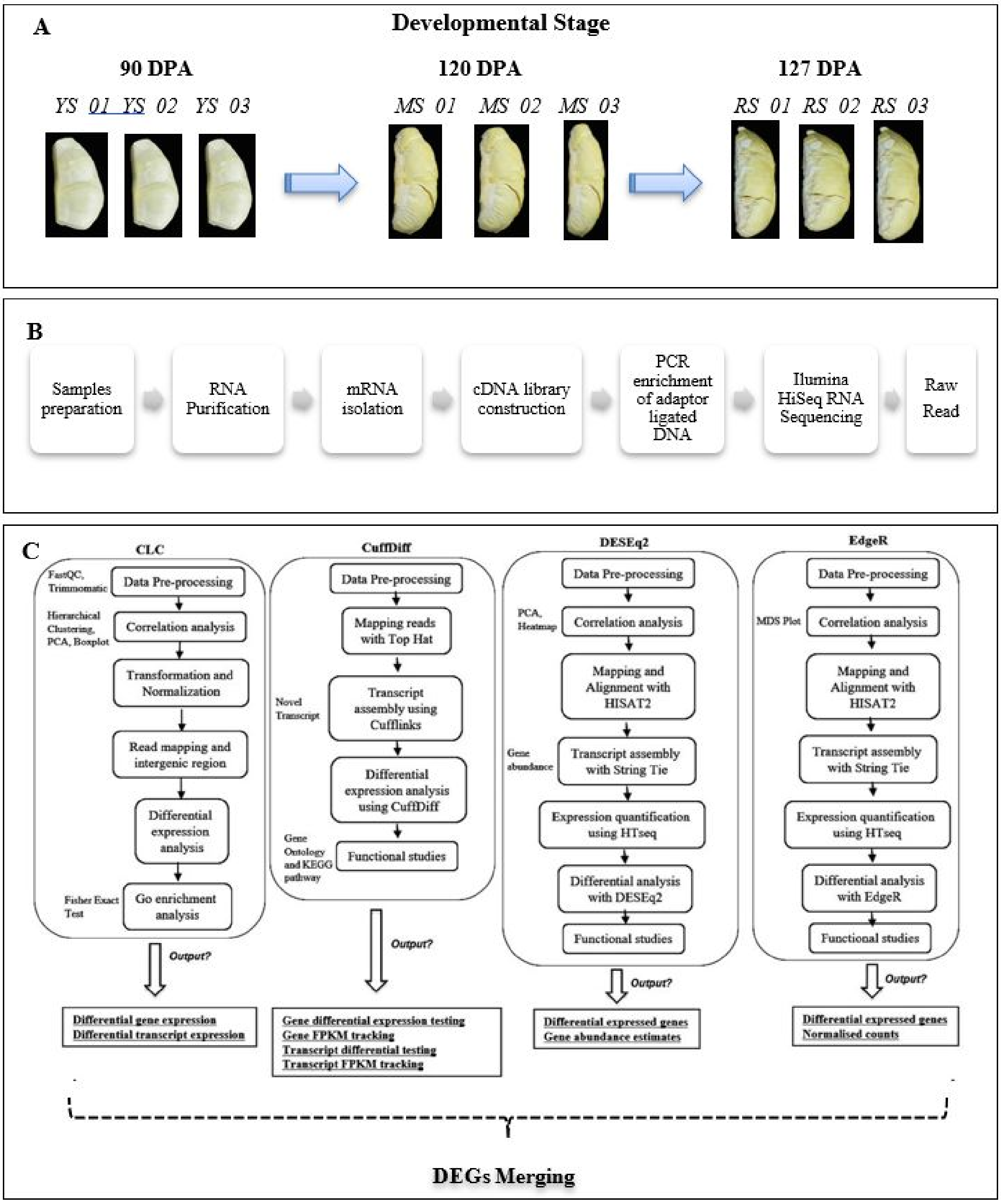


**Figure S1** – Experimental design of the study. (A) The nine samples, a biological triplicate of 90 days post-anthesis (DPA), 120 DPA, and 127 DPA, were used in the transcriptome study. (B) Wet lab experimental workflow, and (C) overview of transcriptome analysis approach using the combined methods.
