# Supplementary material for: Transcriptome analysis during fruit developmental stages in durian (Durio zibethinus Murr.) var. D24
Source: Genet Mol Biol. 2023 Jan 6;45(4):e20210379. doi: 10.1590/1678-4685-GMB-2021-0379 (PMC9830936; doi:10.1590/1678-4685-GMB-2021-0379)
Supplement: Figure S2 - [file 1415-4757-GMB-45-4-e20210379-s2.pdf]

Genetics and Molecular Biology
DOI: https://doi.org/10.1590/1678-4685-GMB-2021-0379

Supplementary Material to "Transcriptome analysis during fruit developmental stages in durian (*Durio zibethinus* Murr.) var. D24"

a

b

c

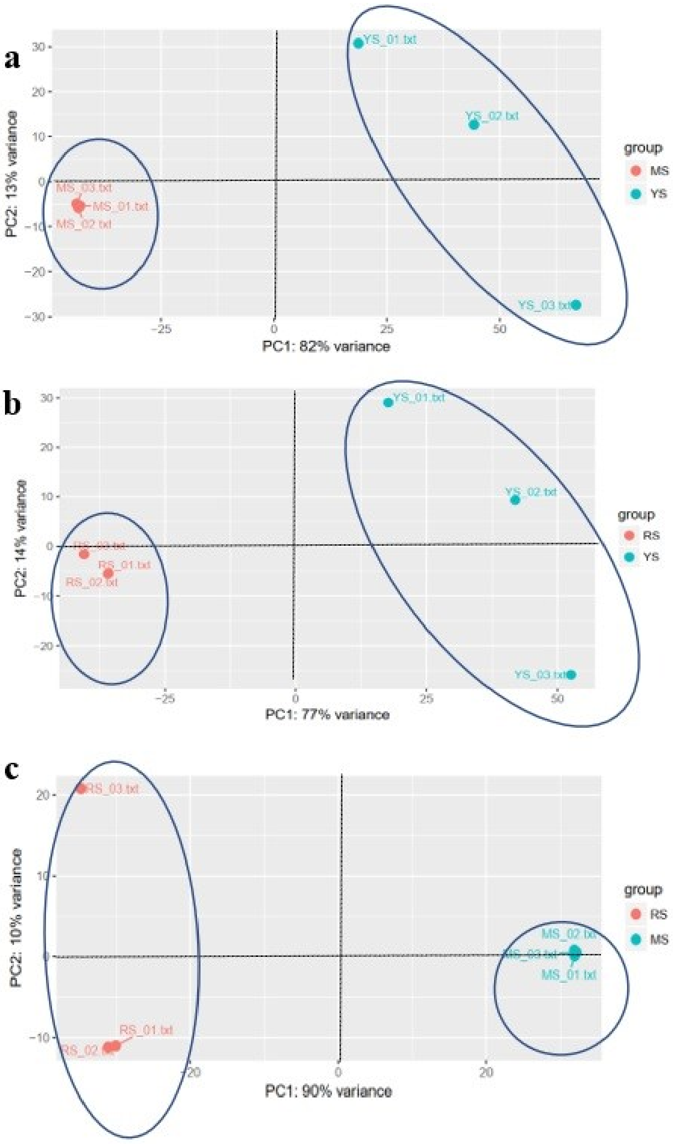

Figure S2 – The comparative Principal component analysis (PCA) plot of the samples at young, mature, and ripening stages. Each replicate was plotted as an individual data point. This type of plot was useful for visualising the overall effect of experimental covariates and batch effects. The percentage of variance indicates how much variance was determined by PC1 and PC2. (a: Young Stage vs Mature Stage (YS/MS), b: Young Stage vs Ripening Stage (YS/RS), c: Mature Stage vs Ripening Stage (MS/RS).
