# Supplementary material for: Transcriptome analysis during fruit developmental stages in durian (Durio zibethinus Murr.) var. D24
Source: Genet Mol Biol. 2023 Jan 6;45(4):e20210379. doi: 10.1590/1678-4685-GMB-2021-0379 (PMC9830936; doi:10.1590/1678-4685-GMB-2021-0379)
Supplement: Table S1 - [file 1415-4757-GMB-45-4-e20210379-s3.pdf]

Genetics and Molecular Biology
DOI: https://doi.org/10.1590/1678-4685-GMB-2021-0379

Supplementary Material to "Transcriptome analysis during fruit developmental stages in durian (*Durio zibethinus* Murr.) var. D24"

Table S1 - Differentially up-regulated expressed genes between the young stage and mature stage of durian fruit pulp. We used FDR: <0.05, Log 2 fold change >1.5 and <-1.5.

Gene Symbol	Gene Name	Log2 fold change	FDR p-value correction
LOC111285147	membrane-anchored ubiquitin-fold protein 3-like	13.3352889	0.04012651
LOC111318243	cytochrome b5-like	12.5541173	0.02234778
LOC111304600	pectinesterase 2-like	11.22367883	8.13E-26
LOC111293884	bZIP transcription factor TGA10-like, transcript	11.20479892	3.42E-25
LOC111298007	probable serine/threonine-protein kinase BSK3	11.1190365	0.00487093
LOC111278395	endoglucanase-like	10.872028	0.02831135
LOC111277078	uncharacterized LOC111277078	10.8154348	0.00101408
LOC111274386	ABC transporter C family member 13	10.7658514	0.01995145
LOC111282227	uncharacterized LOC111282227	10.6329582	0.00557413
LOC111286089	ABC transporter G family member 14-like	10.5499989	0.00515839
LOC111309500	protein ROOT HAIR DEFECTIVE 3 homolog 2-like	10.5240047	0.00034707
LOC111278272	uncharacterized LOC111278272	10.4641515	0.02272886
LOC111315188	allene oxide synthase 1, chloroplastic-like	10.44423	0.000689
MSTRG.31886	Unknown sequences	10.34963	0.005651
LOC111306587	LEAF RUST 10 DISEASE-RESISTANCE LOCUS RECEPTOR-LIKE PROTEIN KINASE-like 2.1	10.25225	0.011144
LOC111299220	uncharacterized LOC111299220	10.16312	1.40E-05
LOC111287425	methionine gamma-lyase-like	10.14481	0.015062
LOC111289620	probable methyltransferase PMT21	10.1332	1.40E-05
LOC111289620	probable methyltransferase PMT21	10.07247152	2.00E-129
LOC111283052	ABSCISIC ACID-INSENSITIVE 5-like protein 7	10.06527	0.000829
LOC111318371	probable serine/threonine-protein kinase At1g01540	10.01212	0.000363
LOC111282902	uncharacterized LOC111282902	10.00101943	4.75E-15
LOC111317091	kinesin-like protein KIN-4A	9.991628	0.001951
LOC111301728	calmodulin-binding protein 60 B-like	9.971501	3.25E-05
LOC111315188	allene oxide synthase 1, chloroplastic-like	9.941797555	2.40E-34
LOC111301330	uncharacterized LOC111301330	9.890983	0.009864

Gene Symbol	Gene Name	Log2 fold change	FDR p-value correction
MSTRG.16320	Unknown sequences	9.884381	0.017358
LOC111287086	probable disease resistance protein At1g58602	9.863055	0.001735
MSTRG.32120	Unknown sequences	9.860302	0.001304
MSTRG.11262	Unknown sequences	9.834361	0.008905
LOC111291064	meiotic recombination protein SPO11-2	9.774328	0.006075
LOC111287487	glucan endo-1,3-beta-glucosidase 11-like	9.762352	0.010156
LOC111297770	DNA-directed RNA polymerase I subunit 1-like	9.740118	0.001338
LOC111302253	uncharacterized membrane protein YuiD-like	9.695723	0.001014
LOC111306077	RNA exonuclease 4	9.694658	0.003033
LOC111299220	uncharacterized LOC111299220	9.688091281	5.40E-32
LOC111303519	SHUGOSHIN 2-like	9.674148	0.013611
LOC111311329	uncharacterized LOC111311329	9.664423	0.000407
LOC111301728	calmodulin-binding protein 60 B-like	9.643236632	5.21E-41
LOC111282399	ELMO domain-containing protein C-like	9.641626	0.01912
LOC111316978	ABC transporter G family member 11-like,	9.632069514	1.60E-36
LOC111305461	serine/threonine-protein kinase OXI1-like	9.61594	0.001961
MSTRG.35109	Unknown sequences	9.602434	0.000342
LOC111296692	nudix hydrolase 26, chloroplastic	9.565827	0.000763
LOC111303365	N-alpha-acetyltransferase 50-like	9.544586	0.009848
LOC111303521	vacuolar protein sorting-associated protein 24 homolog 1	9.527588	0.001597
LOC111283052	ABSCISIC ACID-INSENSITIVE 5-like protein 7	9.52463651	1.04E-29
MSTRG.20171	Unknown sequences	9.522947	6.50E-05
LOC111318157	uncharacterized LOC111318157	9.490975	0.000683
MSTRG.2542	Unknown sequences	9.48254	0.000651
LOC111287285	histidine protein methyltransferase 1 homolog	9.482239	0.024682
LOC111295573	caffeic acid 3-O-methyltransferase-like	9.471809985	1.82E-11
LOC111304634	4-hydroxy-tetrahydrodipicolinate synthase, chloroplastic	9.448434	0.001676
LOC111312385	uncharacterized LOC111312385	9.435338	0.017413
MSTRG.11205	Unknown sequences	9.413073	0.000812
MSTRG.21045	Unknown sequences	9.373216	0.004234
LOC111287086	probable disease resistance protein At1g58602	9.369810051	1.14E-30
LOC111299987	pentatricopeptide repeat-containing protein At5g39350-like	9.314051	0.002189
LOC111289785	probable NADH dehydrogenase [ubiquinone] 1 alpha subcomplex subunit 5, mitochondrial	9.203199	0.037451
TRNAT-UGU	Transfer RNA	9.19047	0.003094
LOC111295509	probable pyridoxal 5'-phosphate synthase subunit PDX1	9.17956	0.011744
MSTRG.32120	Unknown sequences	9.130971593	8.32E-21
LOC111304630	LOB domain-containing protein 18, (Lateral Organ Boundaries)	9.1229	0.000312596
LOC111305461	serine/threonine-protein kinase OXI1-like	9.118252994	9.81E-28

Gene Symbol	Gene Name	Log2 fold change	FDR p-value correction
LOC111304630	LOB domain-containing protein 18	9.103783345	1.35E-12
LOC111274414	post-GPI attachment to proteins factor 3-like	9.09872768	1.36E-34
LOC111298007	probable serine/threonine-protein kinase BSK3	9.03232922	4.49E-13
LOC111286965	uncharacterized LOC111286965	8.980394396	1.46E-09
LOC111303521	vacuolar protein sorting-associated protein 24	8.98022	5.24E-24
LOC111318243	cytochrome b5-like, transcript variant X1	8.977934	4.00E-10
LOC111277078	uncharacterized LOC111277078	8.837425	4.70E-13
MSTRG.31886	Unknown sequences	8.833591	1.00E-12
LOC111312807	tubulin beta-6 chain-like	8.794125	5.65E-29
LOC111275409	un-characterized LOC111275409	8.793297	2.21E-18
LOC111283878	uncharacterized LOC111283878	8.788679046	2.63E-42
LOC111285475	50S ribosomal protein L9, chloroplastic-like,	8.775158	5.65E-61
LOC111283037	homeobox-leucine zipper protein HOX3-like	8.74162798	1.09E-08
LOC111315258	4-coumarate--CoA ligase-like 5	8.649321	1.08E-28
LOC111309500	Protein ROOT HAIR DEFECTIVE 3 homolog 2-like	8.642184	4.11E-13
MSTRG.14226	Unknown sequences	8.590877	1.87E-63
LOC111282227	uncharacterized LOC111282227	8.577669	1.07E-11
LOC111286089	ABC transporter G family member 14-like	8.500941	1.35E-11
LOC111294556	plant cysteine oxidase 2-like, transcript	8.498471955	1.67E-07
LOC111311552	polygalacturonase inhibitor-like	8.43397	0.000312596
MSTRG.21045	Unknown sequences	8.342354	2.22E-13
MSTRG.30203	Unknown sequences	8.338256	2.21E-29
LOC111298473	transcription activator GLK1-like	8.3172436	5.17E-20
LOC111289307	probable serine/threonine-protein kinase PBL15	8.2843	1.01E-14
LOC111295036	intron-binding protein aquarius	8.26521	2.62E-21
MSTRG.4155	Unknown sequences	8.236132	1.24E-39
LOC111274744	uncharacterized LOC111274744	8.227847	1.38E-67
LOC111317678	protein SIEVE ELEMENT OCCLUSION B-like	8.220546	1.61E-14
LOC111318371	probable serine/threonine-protein kinase At1g01540	8.207395	6.69E-12
LOC111293182	ATP-dependent RNA helicase DEAH11, chloroplastic-like	8.196636	4.10E-23
XLOC_006973	novel transcript	8.17641	0.000312596
LOC111313563	uncharacterized LOC111313563	8.14606986	4.12E-07
LOC111301565	beta-(1,2)-xylosyltransferase-like	8.144911	2.40E-42
MSTRG.19557	Unknown sequences	8.13941	7.51E-24
LOC111306587	LEAF RUST 10 DISEASE-RESISTANCE LOCUS RECEPTOR-LIKE PROTEIN KINASE-like 2.1	8.129383	4.56E-09
MSTRG.31589	Unknown sequences	8.076908	8.52E-19
LOC111317091	kinesin-like protein KIN-4A	8.05726	1.50E-10
LOC111287425	methionine gamma-lyase-like	8.048428	1.02E-09
LOC111292651	mavicyanin-like	8.036744907	7.37E-46

Gene Symbol	Gene Name	Log2 fold change	FDR p-value correction
LOC111303063	acyl-CoA-binding protein-like	8.035915981	2.99E-26
LOC111311552	polygalacturonase inhibitor-like	8.023169229	9.88E-18
LOC111295509	probable pyridoxal 5'-phosphate synthase subunit PDX1	7.986618	3.10E-11
LOC111281937	aminotransferase ALD1, chloroplastic-like	7.960043	9.00E-21
LOC111304216	early nodulin-93-like	7.944972454	7.22E-09
MSTRG.7750	Unknown sequences	7.94274	2.91E-36
LOC111276436	ribonuclease 3-like protein 2	7.915384	2.42E-34
LOC111291994	histone-lysine N-methyltransferase setd3-like	7.912995	4.70E-11
LOC111311329	uncharacterized LOC111311329	7.905622	4.00E-11
LOC111313836	K(+) efflux antiporter 2, chloroplastic-like	7.885134	9.14E-22
MSTRG.22577	Unknown sequences	7.872309	1.85E-29
MSTRG.35109	Unknown sequences	7.87218	3.05E-11
LOC111304140	hepatocyte growth factor-regulated tyrosine kinase substrate-like	7.86719	4.89E-14
LOC111297770	DNA-directed RNA polymerase I subunit 1-like	7.854016	4.15E-10
LOC111301330	uncharacterized LOC111301330	7.848045	2.21E-09
LOC111302253	uncharacterized membrane protein YuiD-like	7.840544	2.35E-10
LOC111294297	uncharacterized LOC111294297, transcript variant X3	7.827264502	1.89E-27
LOC111298508	transcription repressor OFP12-like	7.79210963	4.36E-05
LOC111304275	rust resistance kinase Lr10-like	7.786161735	7.72E-06
LOC111307587	uncharacterized LOC111307587	7.677309697	2.63E-32
LOC111292650	triacylglycerol lipase 2-like	7.55833	0.000312596
LOC111276805	uncharacterized LOC111276805	7.548785254	9.00E-17
LOC111309908	cytochrome P450 77A3-like	7.19743169	0.000195223
LOC111293555	benzyl alcohol O-benzoyltransferase-like	7.129697458	5.38E-06
LOC111293555	benzyl alcohol O-benzoyltransferase-like, transcript variant X1	7.10972	0.000312596
LOC111313396	RING-H2 finger protein ATL5-like	6.977802898	5.79E-21
LOC111288096	uncharacterized LOC111288096	6.91513012	0.000315393
LOC111306284	serine/threonine-protein kinase rio2-like	6.897779308	0.000624792
LOC111274286	probable polygalacturonase At3g15720	6.888506571	0.000902289
LOC111276942	non-specific phospholipase C4-like	6.88614166	4.83E-23
LOC111305909	LOB domain-containing protein 19	6.78294307	3.65E-14
LOC111300249	uncharacterized LOC111300249	6.774395536	0.000970999
LOC111281105	protein DETOXIFICATION 40-like	6.68363772	4.17E-38
LOC111306285	protein GLUTAMINE DUMPER 2-like	6.668945983	2.33E-11
LOC111280925	uncharacterized LOC111280925	6.659256641	0.001160978
LOC111290435	LOB domain-containing protein 40-like	6.616392391	2.58E-12
LOC111298611	uncharacterized LOC111298611	6.608186011	0.002060717
LOC111307587	uncharacterized LOC111307587	6.58076	0.00129227
LOC111293921	calmodulin-like	6.571492192	0.000701268
LOC111282509	uncharacterized LOC111282509	6.55632	0.00343537

Gene Symbol	Gene Name	Log2 fold change	FDR p-value correction
LOC111281105	protein DETOXIFICATION 40-like	6.45873	0.000312596
LOC111312958	uncharacterized LOC111312958	6.438727994	7.11E-10
LOC111315580	synaptonemal complex protein 1-like	6.422667564	1.29E-23
LOC111286976	nuclear transcription factor Y subunit C-2-like	6.359892775	0.011187936
LOC111301968	uncharacterized LOC111301968	6.332188541	3.47E-13
LOC111318443	bidirectional sugar transporter N3	6.3118	0.000312596
LOC111280213	cellulose synthase-like protein G2	6.278284098	1.79E-18
LOC111300836	nuclear pore complex protein NUP205-like	6.274587802	0.005580112
LOC111282509	uncharacterized LOC111282509	6.266850539	1.47E-07
LOC111315580	synaptonemal complex protein 1-like	6.25794	0.000312596
LOC111305909	LOB domain-containing protein 19	6.20871	0.00598521
LOC111283141	polygalacturonase QRT3-like	6.200765753	0.002915672
LOC111301538	probable nucleoredoxin 2	6.186988971	1.73E-08
LOC111318443	bidirectional sugar transporter N3	6.165414964	5.10E-06
LOC111301322	ABC transporter G family member 31	6.162000356	2.18E-09
LOC111283759	transcription factor MYB108-like	6.160669576	1.56E-07
LOC111317870	proline-rich receptor-like protein kinase PERK12	6.107352315	2.22E-17
LOC111286614	putative receptor-like protein kinase At3g47110	6.092903782	0.006511795
LOC111276942	non-specific phospholipase C4-like	6.08761	0.00308959
LOC111285498	transmembrane protein 45A-like	6.06232	0.000312596
LOC111290435	LOB domain-containing protein 40-like	6.03502	0.000312596
LOC111285478	protein NRT1/ PTR FAMILY 7.1-like, transcript	6.01832	0.000582785
LOC111282412	LOB domain-containing protein 42-like	6.0083	0.000312596
LOC111301322	ABC transporter G family member 31, transcript	5.95744	0.000312596
LOC111297059	LOB domain-containing protein 1-like	5.65526	0.000312596
LOC111295041	glucan endo-1,3-beta-glucosidase, basic isoform-like	5.60328	0.00614272
LOC111295977	rop guanine nucleotide exchange factor 7-like	5.56923	0.000312596
LOC111316379	aspartic proteinase Asp1-like	5.46952	0.000312596
LOC111296764	probable purple acid phosphatase 20, transcript	5.42737	0.000312596
LOC111301815	transmembrane protein 45A-like	5.41779	0.000312596
LOC111312665	protein DETOXIFICATION 33, transcript variant	5.37174	0.000312596
LOC111312381	uncharacterized methyltransferase At1g78140,	5.3694	0.0214146
LOC111317748	putative SNAP25 homologous protein SNAP30	5.33821	0.000312596
LOC111310483	uncharacterized LOC111310483, transcript variant	5.32815	0.000312596
LOC111311747	ABC transporter G family member 39-like	5.29601	0.000312596
LOC111317870	proline-rich receptor-like protein kinase	5.29238	0.000833693
LOC111304064	probable receptor-like protein kinase At1g80640,	5.28034	0.000312596
LOC111280882	probable 9-cis-epoxycarotenoid dioxygenase	5.21689	0.000312596

Gene Symbol	Gene Name	Log2 fold change	FDR p-value correction
LOC111298913	uncharacterized LOC111298913	5.18175	0.000312596
LOC111313976	uncharacterized LOC111313976	5.17833	0.000312596
LOC111296606	zinc finger protein ZAT12-like	5.17716	0.000312596
LOC111274350	shikimate O-hydroxycinnamoyltransferase-like	5.03821	0.000312596
LOC111290481	hypersensitive-induced reaction 1 protein-like,	5.03038	0.000312596
LOC111287927	cysteine synthase 2-like	5.02663	0.000312596
LOC111304767	uncharacterized LOC111304767	4.94095	0.000312596
LOC111283735	NAC domain-containing protein 100-like	4.86757	0.000312596
LOC111299261	heat stress transcription factor A-2-like,	4.86241	0.000582785
LOC111312254	probable receptor-like serine/threonine-protein	4.85289	0.000312596
LOC111280497	probable potassium transporter 13	4.84641	0.00106839
LOC111309044	UPF0496 protein At4g34320-like, transcript	4.82114	0.000312596
LOC111316857	uncharacterized LOC111316857	4.81577	0.00232875
LOC111312852	tetracycline resistance protein, class H-like,	4.80275	0.000312596
LOC111283869	uncharacterized LOC111283869	4.79708	0.000312596
LOC111276805	uncharacterized LOC111276805, transcript variant	4.79352	0.000312596
LOC111307626	probable serine/threonine-protein kinase WNK5	4.77008	0.000312596
LOC111294332	feruloyl CoA ortho-hydroxylase 1-like	4.75322	0.0338929
XLOC_024860	Novel gene	4.71534	0.00129227
