# Supplementary material for: Transcriptome analysis during fruit developmental stages in durian (Durio zibethinus Murr.) var. D24
Source: Genet Mol Biol. 2023 Jan 6;45(4):e20210379. doi: 10.1590/1678-4685-GMB-2021-0379 (PMC9830936; doi:10.1590/1678-4685-GMB-2021-0379)
Supplement: Table S2 - [file 1415-4757-GMB-45-4-e20210379-s4.pdf]

Genetics and Molecular Biology
DOI: https://doi.org/10.1590/1678-4685-GMB-2021-0379

Supplementary Material to "Transcriptome analysis during fruit developmental stages in durian (*Durio zibethinus* Murr.) var. D24"

Table S2 - Differentially down-regulated expressed genes between the young stage and mature stage of durian fruit pulp. We used FDR: <0.05, Log2 fold change >1.5 and <-1.5.

Gene Symbol	Gene Name	Log2 fold change	FDR p-value correction
MSTRG.34481	Unknown sequences	-5.71934	1.26E-06
LOC111301324	iridoid synthase-like	-5.75332	1.08E-12
LOC111286552	protein PHOTOSYSTEM I ASSEMBLY 2, chloroplastic	-5.75397	1.29E-14
LOC111283752	tubby-like F-box protein 3	-5.77035	6.83E-09
LOC111289347	synaptotagmin-5-like	-5.78155	7.58E-12
MSTRG.17494	Unknown sequences	-5.81282	1.68E-05
MSTRG.9748	Unknown sequences	-5.84638	2.47E-13
LOC111276937	probable cellulose synthase A catalytic subunit 6 [UDP-forming]	-5.85728	3.30E-12
MSTRG.29429	Unknown sequences	-5.89153	9.78E-06
LOC111294072	ubiquitin carboxyl-terminal hydrolase 2-like	-5.89682	1.01E-05
LOC111311962	BTB/POZ and MATH domain-containing protein 3-like	-5.90035	1.11E-05
LOC111314105	proline-rich protein 3-like	-5.94419	9.99E-19
LOC111287804	receptor-like protein 12	-5.97622	3.13E-07
MSTRG.17496	Unknown sequences	-6.01054	5.48E-06
LOC111293582	uncharacterized LOC111293582	-6.0596	4.56E-06
LOC111274855	katanin p60 ATPase-containing subunit A-like 2	-6.08458	1.27E-12
LOC111305908	AT-hook motif nuclear-localized protein 1-like	-6.15924	3.08E-25
LOC111315575	protein ROOT PRIMORDIUM DEFECTIVE 1	-6.21274	1.01E-05
LOC111282083	uncharacterized LOC111282083	-6.2129	2.24E-06
LOC111289814	probable methyltransferase PMT18	-6.25643	1.40E-13
LOC111280314	calcium-dependent protein kinase 21-like	-6.26744	2.29E-78
MSTRG.4787	Unknown sequences	-6.34128	4.11E-14
LOC111292232	pentatricopeptide repeat-containing protein ELI1, chloroplastic	-6.36727	6.84E-07
LOC111292756	UPF0548 protein At2g17695	-6.49622	0.011606
MSTRG.9760	Unknown sequences	-6.53345	0.010832
LOC111304213	protein kinase PINOID 2-like	-6.5739	0.041848

Gene Symbol	Gene Name	Log2 fold change	FDR p-value correction
MSTRG.11548	Unknown sequences	-6.60747	0.010324
LOC111289814	probable methyltransferase PMT18	-6.62664	0.00022
LOC111307584	probable serine/threonine-protein kinase PBL8	-6.62792	6.60E-31
MSTRG.5729	Unknown sequences	-6.62804	7.25E-07
LOC111287804	receptor-like protein 12	-6.63194	0.001394
LOC111291144	protein C2-DOMAIN ABA-RELATED 4-like	-6.64067	0.012722
MSTRG.12961	Unknown sequences	-6.65997	8.47E-13
MSTRG.36427	Unknown sequences	-6.66604	0.010238
LOC111302841	histidine kinase 5-like	-6.68511	1.50E-18
MSTRG.4787	Unknown sequences	-6.69632	0.00031
LOC111283068	phytosulfokine receptor 1-like	-6.74285	2.44E-27
LOC111318002	bidirectional sugar transporter SWEET10-like	-6.74348	0.008228
MSTRG.25731	Unknown sequences	-6.75191	7.58E-10
MSTRG.17492	Unknown sequences	-6.75428	2.37E-13
MSTRG.7090	Unknown sequences	-6.76053	6.66E-08
MSTRG.32710	Unknown sequences	-6.76884	3.72E-27
LOC111304629	uncharacterized LOC111304629	-6.81839	6.04E-08
LOC111307584	probable serine/threonine-protein kinase PBL8	-6.86817	0.000252
MSTRG.2851	Unknown sequences	-6.91285	0.007325
MSTRG.33993	Unknown sequences	-6.95097	4.24E-29
MSTRG.32710	Unknown sequences	-7.01124	0.000202
LOC111302841	histidine kinase 5-like	-7.01672	0.00019
LOC111283068	phytosulfokine receptor 1-like	-7.06065	0.001304
LOC111295507	uncharacterized LOC111295507	-7.07453	0.036571
MSTRG.12961	Unknown sequences	-7.11651	0.000552
MSTRG.33993	Unknown sequences	-7.20243	0.000174
MSTRG.17492	Unknown sequences	-7.20892	0.000183
MSTRG.3263	Unknown sequences	-7.22556	8.31E-16
MSTRG.15890	Unknown sequences	-7.34197	0.004234
LOC111311589	protein trichome birefringence-like	-7.34446	0.00901342
LOC111315804	ethylene-responsive transcription factor	-7.35398	0.0314026
LOC111287953	xyloglucan endotransglucosylase/hydrolase	-7.36103	0.000312596
LOC111279922	calmodulin-like protein 8, transcript variant	-7.3707	0.000312596
LOC111290826	nucleobase-ascorbate transporter 6-like	-7.37673	0.000312596
LOC111293453	3-dehydrosphinganine reductase TSC10A-like	-7.37723	2.57E-72
LOC111318818	mitogen-activated protein kinase kinase kinase 18-like	-7.37864	0.00752884
LOC111278288	calcium permeable stress-gated cation channel	-7.38262	0.000312596

Gene Symbol	Gene Name	Log2 fold change	FDR p-value correction
LOC111281901	transcription factor MYB62-like	-7.38509	0.000582785
LOC111292968	probable xyloglucan endotransglucosylase/hydrolase protein 6	-7.3922	0.0202149
LOC111298258	uncharacterized LOC111298258	-7.39728	0.006276
MSTRG.25731	Unknown sequences	-7.41758	0.000317
MSTRG.17494	Unknown sequences	-7.48989	0.0037
LOC111275658	aspartic proteinase-like	-7.49913	0.000312596
LOC111274739	subtilisin-like protease SBT1.5	-7.53268	0.00397856
LOC111293453	3-dehydrosphinganine reductase TSC10A-like	-7.54033	1.40E-05
MSTRG.29429	Unknown sequences	-7.54618	0.003054
LOC111294072	ubiquitin carboxyl-terminal hydrolase 2-like	-7.56002	0.003163
LOC111311962	BTB/POZ and MATH domain-containing protein 3-like	-7.57949	0.003426
MSTRG.33951	Unknown sequences	-7.5839	2.38E-10
LOC111277773	dehydration-responsive element-binding protein	-7.65162	0.0106799
LOC111283075	transcription factor bHLH162-like	-7.65741	0.0243923
LOC111315575	protein ROOT PRIMORDIUM DEFECTIVE 1	-7.66979	0.00143
MSTRG.17496	Unknown sequences	-7.67053	0.00278
LOC111318712	phospholipase A1-IIbeta-like	-7.68886	0.00192383
MSTRG.3263	Unknown sequences	-7.69625	0.000272
LOC111293487	GEM-like protein 5	-7.71764	0.000312596
LOC111316856	glucose-1-phosphate adenylyltransferase large subunit 1, chloroplastic-like	-7.72275	1.39E-10
LOC111293582	uncharacterized LOC111293582	-7.72921	0.00278
LOC111284733	probable galactinol--sucrose	-7.73393	0.000312596
LOC111314503	fasciclin-like arabinogalactan protein 2	-7.75301	0.012623
LOC111286965	uncharacterized LOC111286965	-7.76867	7.27E-11
LOC111289953	salutaridinol 7-O-acetyltransferase-like	-7.83201	0.000312596
LOC111295285	uncharacterized LOC111295285	-7.89381	1.47E-14
LOC111282083	uncharacterized LOC111282083	-7.89568	0.002551
LOC111293109	AAA-ATPase At3g28610-like	-7.90206	0.00887402
LOC111309924	uncharacterized LOC111309924	-7.916390563	1.92E-39
LOC111309093	protein EXORDIUM-like 2	-7.94134	0.00887402
LOC111292232	pentatricopeptide repeat-containing protein ELI1, chloroplastic	-8.0167	0.001792
LOC111303423	probable xyloglucan endotransglucosylase/hydrolase protein 23	-8.02948	0.000312596
LOC111316185	LOB domain-containing protein 38-like	-8.06646	0.00929446
LOC111305904	axial regulator YABBY 1-like	-8.071802227	2.28E-15
LOC111275659	mitogen-activated protein kinase kinase kinase	-8.08745	0.0106799

Gene Symbol	Gene Name	Log2 fold change	FDR p-value correction
LOC111303287	galactosyltransferase 6	-8.11331	0.000312596
LOC111311549	ras-related protein RABA4d-like	-8.114019028	8.45E-37
MSTRG.5729	Unknown sequences	-8.13011	0.014178
LOC111314766	phosphoprotein ECPP44-like	-8.13335	0.000312596
LOC111278190	stem-specific protein TSJT1-like	-8.16401	0.000312596
LOC111309924	uncharacterized LOC111309924	-8.18371	0.000157
LOC111298174	uncharacterized LOC111298174	-8.29316	0.000312596
LOC111274745	pectinesterase 3	-8.29518	0.000312596
LOC111301563,LOC111301566	glycerophosphodiester phosphodiesterase	-8.3828	0.000833693
MSTRG.7377	Unknown sequences	-8.39974446	8.46E-23
LOC111287471	F-box/kelch-repeat protein At1g67480-like,	-8.40684	0.000312596
MSTRG.7090	Unknown sequences	-8.42358	0.001169
LOC111292380	mitochondrial pyruvate carrier 1-like,	-8.47976	0.000312596
LOC111311549	ras-related protein RABA4d-like	-8.4981	0.00118
LOC111304629	uncharacterized LOC111304629	-8.507	0.001296
LOC111281172	probable WRKY transcription factor 40	-8.51332	0.000312596
LOC111274880	LRR receptor-like serine/threonine-protein	-8.58511	0.000312596
LOC111294535	protein SLE1	-8.58778	0.0361286
LOC111305909	LOB domain-containing protein 19	-8.599375192	1.08E-31
LOC111283076	peroxidase N1-like	-8.62056	0.000312596
LOC111295285	uncharacterized LOC111295285	-8.62972	8.25E-05
LOC111312403	protein EXORDIUM-like	-8.77531	0.000312596
LOC111305904	axial regulator YABBY 1-like	-8.82141	7.15E-05
LOC111290894	abscisic acid 8'-hydroxylase 1-like	-8.83601	0.000312596
MSTRG.7377	Unknown sequences	-8.9350345	3.14E-05
LOC111283603	protein RALF-like 27	-8.992785464	5.35E-27
LOC111316977	uncharacterized LOC111316977	-9.005287613	2.30E-20
LOC111305909	LOB domain-containing protein 19	-9.1192627	0.00206985
LOC111293917	omega-3 fatty acid desaturase,	-9.13208	0.00212999
LOC111315262	L-ascorbate oxidase homolog	-9.13668	0.0130188
LOC111316936	cytochrome P450 CYP736A12-like	-9.21472	0.0193685
LOC111284909	asparagine synthetase [glutamine-hydrolyzing] 1	-9.21616	0.00343537
LOC111312815	chaperone protein dnaJ 11, chloroplastic-like	-9.25731	0.038914
MSTRG.33951	Unknown sequences	-9.2749643	0.00041031
LOC111303526	heavy metal-associated	-9.28425	0.000312596
LOC111281364	dehydration-responsive element-binding protein 1A-like	-9.37327	0.000582785
LOC111316856	glucose-1-phosphate adenylyltransferase large subunit 1, chloroplastic-like	-9.4525362	0.0004438
LOC111286965	uncharacterized LOC111286965	-9.4810942	0.00035447

Gene Symbol	Gene Name	Log2 fold change	FDR p-value correction
LOC111283603	protein RALF-like 27	-9.5559029	2.18E-05
LOC111288324	UDP-glucuronate 4-epimerase 6-like	-9.65161	0.00451342
LOC111295419	protein CDI-like	-9.7041	0.00999511
MSTRG.31294	Unknown sequences	-9.711706002	1.28E-17
LOC111305464	auxin-induced in root cultures protein 12-like	-9.74314	0.0108235
LOC111309042	transcription termination factor MTEF1, chloroplastic	-9.800410409	5.82E-25
LOC111316977	uncharacterized LOC111316977	-9.8066878	3.34E-05
LOC111304131	uncharacterized LOC111304131	-10.19820124	1.86E-07
LOC111294115	protein NUCLEAR FUSION DEFECTIVE 4-like	-10.21194632	0.000279608
LOC111308224	actin-depolymerizing factor 5	-10.24748068	2.13E-10
LOC111299223	E3 ubiquitin-protein ligase ATL4-like	-10.28077601	1.03E-47
LOC111291309	abscisate beta-glucosyltransferase-like	-10.2951	0.000312596
LOC111318679	GDSL esterase/lipase At1g29670-like	-10.29761004	1.93E-07
LOC111284187	ACT domain-containing protein ACR2	-10.29959505	8.68E-08
LOC111310206	triosephosphate isomerase, cytosolic	-10.3003751	1.81E-10
LOC111308606	cytochrome P450 CYP82D47-like	-10.30891723	1.89E-28
LOC111308711	expansin-like A2	-10.31275919	7.64E-16
LOC111292555	probable galacturonosyltransferase 15	-10.3326013	2.62E-16
LOC111287561	hydroquinone glucosyltransferase-like	-10.33795766	3.53E-09
LOC111289734	protein STICHEL-like 2	-10.34842623	0.000478496
LOC111305464	auxin-induced in root cultures protein 12-like	-10.3510225	1.11E-26
LOC111303366	probable boron transporter 6	-10.38437493	1.35E-10
LOC111296264	galactinol synthase 2-like	-10.47539103	2.11E-18
LOC111280367	sugar carrier protein C-like	-10.4941838	6.74E-05
LOC111301331	uncharacterized LOC111301331	-10.49676028	1.34E-11
LOC111295419	protein CDI-like	-10.5042274	6.30E-60
LOC111318057	MLP-like protein 43	-10.50850491	4.08E-12
LOC111291687	3-ketoacyl-CoA synthase 11-like	-10.56048853	7.26E-09
LOC111274387	probable WRKY transcription factor 40	-10.60567034	1.59E-11
LOC111318371	probable serine/threonine-protein kinase At1g01540	-10.63722478	6.63E-17
LOC111306117	protein DETOXIFICATION 49-like	-10.65290621	8.57E-07
LOC111285821	12-oxophytodienoate reductase 3-like	-10.65437297	4.17E-08
LOC111315188	allene oxide synthase 1, chloroplastic-like	-10.662	0.0496018
LOC111285984	glucomannan 4-beta-mannosyltransferase 2-like	-10.66770154	4.73E-07
LOC111309042	transcription termination factor MTEF1, chloroplastic	-10.673562	1.69E-05
LOC111317093	ethylene-responsive transcription factor RAP2-10-like	-10.69811349	1.43E-16
LOC111311239	WAT1-related protein At1g21890-like	-10.70063849	1.42E-07
LOC111274892	probable WRKY transcription factor 28	-10.7513683	6.14E-05

Gene Symbol	Gene Name	Log2 fold change	FDR p-value correction
LOC111278395	endoglucanase-like	-10.80853179	1.31E-10
LOC111316161	transcription factor bHLH18-like	-10.84843791	9.30E-08
LOC111301786	beta-amyrin 28-oxidase-like	-10.88131029	2.40E-07
LOC111290490	ethylene-responsive transcription factor	-10.93766998	2.98E-19
LOC111290758	bidirectional sugar transporter SWEET7-like	-10.995443	5.40E-10
LOC111295502	GATA transcription factor 8-like, transcript	-11.0041165	5.26E-09
LOC111285778	LOB domain-containing protein 12	-11.01957649	1.50E-05
LOC111278395	endoglucanase-like	-11.0334	0.000833693
LOC111289922	transcription factor bHLH162-like	-11.08706906	1.13E-08
LOC111290676	monocopper oxidase-like protein SKU5	-11.14908047	5.63E-08
LOC111279835	inorganic phosphate transporter 1-4-like	-11.16340772	1.34E-05
LOC111314108	probable pectinesterase/pectinesterase inhibitor	-11.1741725	7.75E-08
LOC111314312	inorganic phosphate transporter 1-4-like	-11.19136459	1.08E-05
LOC111308606	cytochrome P450 CYP82D47-like	-11.214392	1.69E-05
LOC111309796	CASP-like protein 4D1	-11.24042127	3.13E-23
LOC111315188	allene oxide synthase 1, chloroplastic-like	-11.32095844	3.09E-32
LOC111275649	glucan endo-1,3-beta-glucosidase 12-like	-11.43236842	4.59E-10
MSTRG.31294	Unknown sequences	-11.594121	6.05E-05
LOC111284125	protein P21-like	-11.67915157	5.99E-07
LOC111296083	uncharacterized protein At1g04910-like,	-11.78699476	1.34E-21
LOC111285489	ethylene-responsive transcription factor TINY-like	-11.83986588	5.35E-09
LOC111291469	protein NRT1/ PTR FAMILY 2.11-like	-12.05371469	7.75E-08
LOC111281964	receptor-like protein kinase HAIKU2	-12.54736279	4.72E-11
LOC111318240	protein NRT1/ PTR FAMILY 5.5-like	-13.11914601	1.44E-21
LOC111289849	bidirectional sugar transporter SWEET10-like	-13.95054439	2.49E-18
LOC111275112	sugar carrier protein C	-14.35919627	5.18E-08
