# Supplementary material for: Transcriptome analysis during fruit developmental stages in durian (Durio zibethinus Murr.) var. D24
Source: Genet Mol Biol. 2023 Jan 6;45(4):e20210379. doi: 10.1590/1678-4685-GMB-2021-0379 (PMC9830936; doi:10.1590/1678-4685-GMB-2021-0379)
Supplement: Table S3 - [file 1415-4757-GMB-45-4-e20210379-s5.pdf]

Genetics and Molecular Biology
DOI: https://doi.org/10.1590/1678-4685-GMB-2021-0379

Supplementary Material to "Transcriptome analysis during fruit developmental stages in durian (*Durio zibethinus* Murr.) var. D24"

Table S3 - Differentially up-regulated expressed genes between the mature stage and ripening stage of durian fruit pulp. We used FDR: <0.05, Log2 fold change >1.5 and <-1.5.

Gene Symbol	Gene Name	Log2 fold change	FDR p-value correction
LOC111295531	glucan endo-1,3-beta-glucosidase, basic	11.12739807	2.40E-13
LOC111304243	uncharacterized LOC111304243	10.82842026	3.19E-37
LOC111304213	protein kinase PINOID 2-like	10.81391	2.20E-09
LOC111316688	probable leucine-rich repeat receptor-like	10.73250792	3.19E-36
LOC111316289	probable leucine-rich repeat receptor-like	10.18733519	1.32E-24
LOC111304213	protein kinase PINOID 2-like	10.10328474	4.43E-91
LOC111296590	glutathione S-transferase U8-like	9.819365319	2.58E-65
LOC111283910	uncharacterized protein At1g04910-like,	9.624435594	7.93E-23
LOC111299988	ethylene-responsive transcription factor	9.575002879	1.25E-20
LOC111305012	beta-amylase 3, chloroplastic-like	9.545033365	7.88E-24
LOC111274639	protein indeterminate-domain 7-like, transcript	9.538087376	1.35E-13
LOC111303682	17.3 kDa class I heat shock protein-like	9.526186953	4.23E-21
LOC111317014	probable mannitol dehydrogenase	9.51845	0.0167276
LOC111311521	polygalacturonase-like	9.48951	0.00020799
MSTRG.23678	Unknown sequences	9.468612	2.00E-12
LOC111312780	probable LRR receptor-like serine/threonine-protein kinase At4g36180	9.419361796	1.65E-11
LOC111318053	WUSCHEL-related homeobox 6-like	9.392742016	5.40E-20
LOC111277545	formin-like protein 16	9.375942033	6.21E-12
LOC111292776	transcription factor HEC3-like	9.346581074	2.57E-10
LOC111275124	zinc finger protein CONSTANS-LIKE 4-like,	9.272614704	9.57E-18
LOC111282979	auxin efflux carrier component 7-like	9.247544358	2.63E-07
LOC111288554	uncharacterized LOC111288554, transcript variant X1	9.221445682	2.02E-13
LOC111311996	laccase-7-like	9.168182881	1.54E-10
LOC111311521	polygalacturonase-like	9.166818974	1.67E-24
LOC111306867	putative E3 ubiquitin-protein ligase LIN-1,	9.119078894	1.17E-14
LOC111276419	fasciclin-like arabinogalactan protein 1	9.103103988	2.27E-13
LOC111311224	probable calcium-binding protein CML44	9.097227318	1.41E-20

Gene Symbol	Gene Name	Log2 fold change	FDR p-value correction
LOC111276733	uncharacterized LOC111276733	9.064677	9.26E-08
LOC111294224	hevein-like preproprotein	9.039798581	6.52E-11
LOC111278395	endoglucanase-like	9.03817	0.00206807
LOC111317014	probable mannitol dehydrogenase	9.018448275	5.03E-28
LOC111291461	putative calcium-binding protein CML19	9.008240216	6.76E-13
LOC111318057	MLP-like protein 43	8.970261229	1.38E-09
LOC111300619	uncharacterized protein LOC111300619 isoform X1	8.930274519	2.60E-08
LOC111308606	cytochrome P450 CYP82D47-like	8.906831	4.60E-13
LOC111282766	transcription factor TCP8-like	8.884562	1.29E-07
LOC111295536	exocyst complex component EXO70H1-like	8.82363	0.00516878
LOC111317417	probable xyloglucan endotransglucosylase/hydrolase protein 33	8.793108152	1.17E-06
LOC111287629	small heat shock protein, chloroplastic-like	8.76183	0.00727459
LOC111278348	uncharacterized protein LOC111278348	8.725124166	1.15E-83
LOC111278395	endoglucanase-like	8.694660578	4.63E-126
LOC111296235	abscisic acid 8'-hydroxylase 4-like isoform X1	8.664260631	3.16E-25
LOC111294546	hsp70 nucleotide exchange factor fes1-like	8.65954	8.86E-14
LOC111306385	LOW QUALITY PROTEIN: aluminum-activated malate transporter 10-like	8.605369446	1.53E-21
LOC111311549	ras-related protein RABA4d-like	8.605315	8.62E-14
LOC111295536	xocyst complex component EXO70H1-like	8.539212156	8.88E-55
LOC111298613	equilibrative nucleotide transporter 3-like	8.494437461	3.89E-09
LOC111307281	crocetin glucosyltransferase, chloroplastic-like	8.492268702	2.16E-09
LOC111283003	Citrate regulatory gene2 protein-like isoform X1	8.425474473	5.68E-15
LOC111275649	glucan endo-1,3-beta-glucosidase 12-like	8.393470393	6.00E-06
LOC111285630	copper transporter 1-like	8.38687971	1.24E-07
LOC111294910	arabinogalactan peptide 13-like	8.380379689	2.07E-07
LOC111294546	hsp70 nucleotide exchange factor fes1-like	8.368659227	4.37E-115
LOC111296858	glutathione S-transferase U8-like	8.363805553	8.88E-57
LOC111300662	stress enhanced protein 2, chloroplastic-like	8.34376	0.0435014
LOC111311549	ras-related protein RABA4d-like	8.310783961	1.74E-113
LOC111285272	uncharacterized protein LOC111285272	8.298631469	5.68E-101
LOC111295761	uncharacterized protein LOC111295761	8.276028728	5.77E-10
LOC111304696	cinnamoyl-CoA reductase-like SNL6	8.238662814	9.14E-08
LOC111305934	actin-depolymerizing factor 10	8.223795205	6.37E-14
LOC111308606	cytochrome P450 CYP82D47-like	8.203264708	4.73E-48
LOC111302013	BURP domain protein RD22-like	8.195761277	3.18E-36
LOC111299025	heptahelical transmembrane protein 1-like isoform X1	8.16854674	4.73E-36
LOC111300662	stress enhanced protein 2, chloroplastic-like	8.140500837	3.42E-36
LOC111316166	probable xyloglucan endotransglucosylase/hydrolase protein 6	8.13317405	1.00E-05

**Gene Symbol**	**Gene Name**	**Log2 fold change**	**FDR p-value correction**
LOC111285251	protein LURP-one-related 8-like	8.127684788	1.90E-08
LOC111274209	uncharacterized protein LOC111274209	8.11798426	1.50E-22
LOC111312913	probable serine/threonine-protein kinase PIX13	8.009359	5.15E-13
LOC111291309	abscisate beta-glucosyltransferase-like	7.96966	0.00020799
LOC111280333	pseudogene	7.965274278	1.32E-27
LOC111293048	probable Histone-lysine N-methyltransferase ATXR5	7.899614	5.53E-06
LOC111278572	inorganic pyrophosphatase 2-like	7.82673	0.00020799
LOC111311985	glutathione S-transferase U8-like	7.71176	0.00020799
LOC111285272	uncharacterized LOC111285272	7.67617	0.00287618
LOC111293717	S-adenosylmethionine synthase 3-like	7.630571	5.98E-05
LOC111287154	sodium/calcium exchanger NCL-like	7.62475	0.0160447
LOC111305387	sm-like protein LSM5	7.466335	3.86E-05
LOC111312192	probable choline kinase 1, transcript variant	7.45644	0.00020799
LOC111312913	probable serine/threonine-protein kinase PIX13	7.448223943	2.76E-43
MSTRG.33739	Unknown sequences	7.375775	6.57E-10
LOC111310012	putative disease resistance protein At3g14460	7.272589	7.62E-05
MSTRG.5729	Unknown sequences	7.237462	1.97E-12
LOC111311763	pentatricopeptide repeat-containing protein At5g12100, mitochondrial-like	7.234828	6.26E-05
LOC111294072	ubiquitin carboxyl-terminal hydrolase 2-like	7.229179	7.16E-05
MSTRG.29429	Unknown sequences	7.213589	6.82E-05
LOC111288064	protein WVD2-like 4, transcript variant X1	7.19024	0.00926099
LOC111294224	hevein-like preproprotein	7.13727	0.016141
MSTRG.5729	Unknown sequences	7.068946481	3.11E-196
LOC111298258	uncharacterized LOC111298258	7.061663	0.000349
LOC111299242	17.1 kDa class II heat shock protein-like	7.03782	0.00039263
MSTRG.15890	Unknown sequences	7.007487	0.000163
MSTRG.36117	Unknown sequences	6.985456	1.41E-08
LOC111293487	GEM-like protein 5	6.91324	0.00020799
MSTRG.18936	Unknown sequences	6.905658	0.000288
LOC111311546	OTU domain-containing protein At3g57810-like	6.87995	0.000378
LOC111274057	NADH-ubiquinone oxidoreductase 20.9 kDa subunit-like	6.868571	0.000225
LOC111282845	probable nucleoredoxin 1	6.86519	0.00020799
LOC111303701	17.5 kDa class I heat shock protein-like	6.73551	0.00020799
LOC111295774	zinc finger protein ZAT10-like	6.71977	0.00020799
LOC111287951	NADH-ubiquinone oxidoreductase 20.9 kDa subunit-like	6.71362	6.67E-07
LOC111318631	dormancy-associated protein homolog 4-like	6.70525	0.0184336
LOC111303683	7.3 kDa class I heat shock protein-like	6.69473	0.028259
LOC111292230	crocetin glucosyltransferase,	6.59706	0.00670448

Gene Symbol	Gene Name	Log2 fold change	FDR p-value correction
LOC111305070	beta-glucosidase 11-like	6.58271	0.0424588
LOC111296619	glutathione S-transferase U8-like	6.53719	0.00020799
MSTRG.12961	Unknown sequences	6.516409	7.07E-09
LOC111276049	uncharacterized LOC111287951	6.511039	0.000795
LOC111287951	uncharacterized LOC111287951	6.505450314	9.18E-90
LOC111304849	uncharacterized protein At4g00950-like	6.488541	4.31E-07
LOC111294289	protein STRICTOSIDINE SYNTHASE-LIKE 10-like	6.480273	0.000765
MSTRG.33739	Unknown sequences	6.453038713	9.34E-20
LOC111288059	uncharacterized LOC111288059	6.44202	0.0178791
LOC111318002	bidirectional sugar transporter SWEET10-like	6.411296	0.000958
LOC111308755	protein EARLY-RESPONSIVE TO DEHYDRATION 7,	6.40309	0.00020799
MSTRG.4785	Unknown sequences	6.395721	1.07E-12
LOC111306845	uncharacterized LOC111306845	6.392457	0.001692
LOC111288524	probable glutathione S-transferase	6.34518	0.00020799
TRNAG-CCC	Transfer RNA	6.298628	6.36E-10
LOC111277539	protein PHR1-LIKE 1-like	6.29326	3.13E-11
LOC111283603	protein RALF-like 27	6.292572	3.74E-12
LOC111274403	BRCA1-associated protein-like	6.254206	0.001801
LOC111279066	ankyrin repeat-containing protein	6.24248	0.00104814
LOC111282160	homeobox-leucine zipper protein HAT5-like	6.23377	5.44E-11
MSTRG.4785	Unknown sequences	6.221518293	1.79E-107
LOC111303688	class I heat shock protein-like	6.20333	0.00020799
LOC111287988	protein SRC2-like	6.19765	0.00020799
LOC111286287	uncharacterized LOC111286287	6.196615	0.001955
LOC111276733	uncharacterized LOC111276733	6.189345	8.78E-12
LOC111308603	cytochrome P450 82C4-like	6.161448	0.003852
MSTRG.14611	Unknown sequences	6.146772	0.002613
LOC111292968	probable xyloglucan	6.1364	0.0303538
LOC111304865	pentatricopeptide repeat-containing protein At5g16420, mitochondrial	6.10152	8.79E-08
LOC111283809	protein VACUOLELESS1-like	6.101143	0.002969
MSTRG.36117	Unknown sequences	6.076991	3.55E-17
LOC111287953	xyloglucan endotransglucosylase/hydrolase	6.05873	0.00020799
MSTRG.25731	Unknown sequences	6.051906	3.17E-11
LOC111296917	F-box/LRR-repeat protein 3-like	6.04753	0.0069325
LOC111283603	protein RALF-like 27	6.033307	1.36E-50
LOC111311009	UDP-glycosyltransferase 73C6-like	6.02147	0.0179651
LOC111315694	uncharacterized LOC111315694	6.021064	0.003436
MSTRG.32862	Unknown sequences	6.017646	0.003221
LOC111300451	VQ motif-containing protein 31-like	6.00263	0.0320016
LOC111298889	probable glutathione S-transferase	6.00115	0.00020799

Gene Symbol	Gene Name	Log2 fold change	FDR p-value correction
LOC111300248	uncharacterized LOC111300248	6.000248	0.003944
TRNAE-CUC	Transfer RNA	5.995896	1.36E-12
LOC111306167	uncharacterized LOC111306167	5.99026	0.00020799
LOC111282160	homeobox-leucine zipper protein HAT5-like	5.971007	2.87E-48
MSTRG.25133	Unknown sequences	5.967606	0.003794
LOC111310303	MLO-like protein 6	5.9592	0.005599
LOC111285576	chitotriosidase-1-like	5.9544	0.00553144
LOC111274382	putative 12-oxophytodienoate reductase 11	5.94491	0.00860083
LOC111293969	phosphoenolpyruvate carboxylase kinase 1-like, transcript variant X1	5.92943	0.00020799
LOC111303611	zinc finger protein ZAT12-like	5.92177	0.00020799
LOC111277539	protein PHR1-LIKE 1-like	5.902129	4.76E-30
LOC111284075	probable disease resistance protein At4g27220	5.899992	0.004545
MSTRG.22660	LOC111278267	5.896968	1.41E-08
LOC111296955	E3 ubiquitin-protein ligase ATL31-like	5.89603	0.00020799
LOC111277190	perakine reductase-like	5.87857	0.0013488
LOC111316515	GEM-like protein 5	5.86146	0.00020799
MSTRG.15123	Unknown sequences	5.852701	0.000146
LOC111286661	uncharacterized LOC111286661	5.83691	0.018531
TRNAE-CUC	Transfer RNA	5.830207	2.46E-80
LOC111307193	probable WRKY transcription factor 70	5.82317	0.00119934
LOC111316628	probable galactinol--sucrose	5.8137	0.00056455
LOC111313028	BAG family molecular chaperone regulator 6-like	5.81305	0.0179651
LOC111312788	uncharacterized LOC111312788	5.80826	0.00020799
LOC111290564	vignain-like	5.7863	0.00020799
TRNAG-CCC	Transfer RNA	5.717676	1.76E-19
MSTRG.12961	Unknown sequences	5.650746	4.26E-14
MSTRG.15123	Unknown sequences	5.454912	1.08E-28
LOC111278267	uncharacterized LOC111278267	5.345298	1.06E-16
MSTRG.34120	Unknown sequences	5.336981	5.35E-43
LOC111304865	pentatricopeptide repeat-containing protein At5g16420, mitochondrial	5.258758	6.16E-12
MSTRG.6601	Unknown sequences	5.239326	4.26E-25
LOC111304849	uncharacterized protein At4g00950-like	5.239088	1.59E-09
LOC111317018	protein BRICK 1	5.206922	4.63E-18
LOC111289594	transcription factor MYBS1-like	5.19537	4.68E-45
LOC111293048	probable Histone-lysine N-methyltransferase ATXR5	5.169879	1.05E-07
LOC111301699	thioredoxin-like protein CDSP32, chloroplastic	5.082786	1.09E-12
LOC111308611	transmembrane protein 45B	5.007227	4.88E-64
LOC111275914	probable L-type lectin-domain containing receptor kinase V.3	4.993838	2.39E-10

Gene Symbol	Gene Name	Log2 fold change	FDR p-value correction
LOC111275243	probable carboxylesterase SOBER1-like	4.946835	1.17E-27
LOC111277239	14 kDa proline-rich protein DC2.15-like	4.943081	2.03E-20
MSTRG.31257	Unknown sequences	4.930831	5.57E-21
LOC111314105	proline-rich protein 3-like	4.903135	1.90E-22
LOC111294357	sulfate transporter 1.3-like	4.88543	9.62E-26
LOC111287737	G-type lectin S-receptor-like serine/threonine-protein kinase At4g27290	4.884873	7.88E-13
LOC111283194	protein STRUBBELIG-RECEPTOR FAMILY 3-like	4.880131	7.65E-37
LOC111286965	uncharacterized LOC111286965	4.813336	3.98E-12
MSTRG.23594	Unknown sequences	4.807502	6.05E-42
MSTRG.34847	Unknown sequences	4.764986	3.74E-06
MSTRG.2344	Unknown sequences	4.724727	8.10E-42
LOC111305387	sm-like protein LSM5	4.724291	3.39E-06
LOC111285741	glucan endo-1,3-beta-glucosidase	4.603205	7.69E-25
LOC111311763	pentatricopeptide repeat-containing protein At5g12100, mitochondrial-like	4.551911	9.29E-06
LOC111310012	putative disease resistance protein At3g14460	4.541002	1.14E-05
MSTRG.29429	Unknown sequences	4.530304	1.07E-05
LOC111294072	ubiquitin carboxyl-terminal hydrolase 2-like	4.527706	1.14E-05
LOC111293557	nuclear pore complex protein NUP1-like	4.502059	1.66E-06
MSTRG.3089	Unknown sequences	4.463789	1.43E-59
