# Supplementary material for: Transcriptome analysis during fruit developmental stages in durian (Durio zibethinus Murr.) var. D24
Source: Genet Mol Biol. 2023 Jan 6;45(4):e20210379. doi: 10.1590/1678-4685-GMB-2021-0379 (PMC9830936; doi:10.1590/1678-4685-GMB-2021-0379)
Supplement: Table S4 - [file 1415-4757-GMB-45-4-e20210379-s6.pdf]

Genetics and Molecular Biology
DOI: https://doi.org/10.1590/1678-4685-GMB-2021-0379

Supplementary Material to "Transcriptome analysis during fruit developmental stages in durian (*Durio zibethinus* Murr.) var. D24"

Table S4 - Differentially down-regulated expressed genes between the mature stage and ripening stage of durian fruit pulp. We used FDR: <0.05, Log2 fold change >1.5 and <-1.5.

Gene Symbol	Gene Name	Log2 fold change	FDR p-value correction
LOC111309322	sterol 14-demethylase-like	-3.95444	0.0005645
LOC111294891	uncharacterized LOC111294891	-3.98419	0.00020799
LOC111316121	inositol-3-phosphate synthase-like	-4.03379	0.0369311
LOC111277257	uncharacterized LOC111277257, transcript variant	-4.04538	0.00020799
LOC111316987	homeobox-leucine zipper protein ATHB-6-like	-4.05321	0.00020799
LOC111277014	plant cysteine oxidase 2-like, transcript	-4.06902	0.00020799
LOC111315252, LOC111315261	peroxisome biogenesis protein 19-2-like, protein transport protein SEC16B homolog	-4.07442	0.00056455
LOC111302215	zinc transporter 6, chloroplastic-like	-4.077	0.00020799
LOC111281105	protein DETOXIFICATION 40-like	-4.10876	0.00020799
LOC111299368	ras-related protein Rab7-like	-4.19375	0.00149434
LOC111279856	protein MARD1-like, transcript variant X1	-4.21019	0.00020799
LOC111275775	uncharacterized LOC111275775	-4.22938	0.00020799
LOC111296642	DExH-box ATP-dependent RNA helicase DExH12-like	-4.24855	0.00727459
LOC111297335	trihelix transcription factor PTL-like	-4.25548	0.00020799
LOC111294297	uncharacterized LOC111294297, transcript variant	-4.25692	0.00020799
LOC111299310	probably inactive leucine-rich repeat	-4.26403	0.00020799
LOC111289583	protein DOWNY MILDEW RESISTANCE 6-like	-4.31068	0.00020799
LOC111299532	kelch repeat-containing protein At3g27220-like,	-4.32275	0.00020799
LOC111301337	laccase-4-like	-4.33192	0.00882146
LOC111297909	uncharacterized LOC111297909	-4.38582	0.00020799
LOC111314020	protein S-acyltransferase 18-like, transcript	-4.39634	0.00020799
LOC111306227	uncharacterized LOC111306227	-4.47374	0.0369311
LOC111285675	leucine-rich repeat receptor-like protein kinase	-4.53732	0.00020799
LOC111277427	basic proline-rich protein-like	-4.6469	0.00020799
LOC111289856	traB domain-containing protein, transcript	-4.70252	0.00020799
LOC111311845	cytokinin riboside 5'-monophosphate	-4.79953	0.0153939

Gene Symbol	Gene Name	Log2 fold change	FDR p-value correction
LOC111316168	transcription factor RAX3-like	-4.81611	0.00020799
LOC111316158	uncharacterized protein At5g65660-like	-5.04522	0.00119934
LOC111282836	glutamate formimidoyltransferase-like	-5.05711	0.00020799
LOC111285374	probable indole-3-pyruvate monooxygenase YUCCA4	-5.09951	0.00020799
LOC111274050	uncharacterized LOC111274050	-5.13378	0.00020799
LOC111294421	aquaporin SIP1-1-like	-5.21543	0.00020799
LOC111288008	uncharacterized LOC111288008	-5.32271	0.0153939
LOC111313328	uncharacterized LOC111313328	-5.3437	0.00020799
LOC111280755	uncharacterized LOC111280755	-5.36599	0.00020799
LOC111313396	RING-H RING-H2 finger protein ATL5-like2 finger protein ATL5-like	-5.42314	0.00149434
LOC111294357	sulfate transporter 1.3-like, transcript variant	-5.4384	0.00020799
LOC111301699	thioredoxin-like protein CDSP32, chloroplastic	-5.46366	0.0444584
LOC111277239	14 14 kDa proline-rich protein DC2.15-like kDa proline-rich protein DC2.15-like	-5.55792	0.00491837
LOC111302352	zinc finger protein 2-like	-5.82013	0.00020799
LOC111296030	protein PIN-LIKES 3-like	-5.915014811	0.001385095
LOC111299239	solute carrier family 25 member 44-like	-5.92814	0.000208
LOC111296829	uncharacterized protein LOC111296829	-5.951010534	0.00347361
LOC111297280	fructose-bisphosphate aldolase 1, chloroplastic-like	-5.95271	0.000208
LOC111307200	non-specific lipid-transfer protein 3-like	-5.957471163	0.000736369
LOC111296871	serine carboxypeptidase-like 27 isoform X1	-5.97343927	0.002488342
LOC111311845	cytokinin riboside 5'-monophosphate phosphoribohydrolase LOG3-like	-5.98316846	1.71E-23
LOC111311241	WAT1-related protein At4g08300-like	-6.004389786	0.000324017
LOC111276733	uncharacterized protein LOC111276733	-6.033095468	5.32E-22
LOC111298691	classical arabinogalactan protein 9-like	-6.039672988	0.00050363
LOC111277115	uncharacterized LOC111277115	-6.04314	0.000208
LOC111286976	nuclear transcription factor Y subunit C-2-like	-6.056477887	0.005249413
LOC111297709	uncharacterized LOC111297709	-6.06598	0.0431503
LOC111283878	uncharacterized protein LOC111283878	-6.155812456	9.12E-67
LOC111313342	myb-related protein 308-like	-6.15936	0.0211934
LOC111299239	solute carrier family 25 member 44-like	-6.160903654	2.22E-69
LOC111281093	uncharacterized protein LOC111281093	-6.218754239	0.003594237
LOC111288040	ethylene receptor-like	-6.21884	3.05E-164
LOC111302663	17.3 kDa class I heat shock protein-like	-6.22236	4.59E-26
LOC111289805	mavicyanin-like	-6.24476	2.96E-13

Gene Symbol	Gene Name	Log2 fold change	FDR p-value correction
LOC111310679	uncharacterized protein LOC111310679	-6.254454744	0.000407877
MSTRG.20794	Unknown sequences	-6.25521	5.49E-13
LOC111299987	pentatricopeptide repeat-containing protein At5g39350-like	-6.29017	2.05E-11
LOC111296451	uncharacterized LOC111296451	-6.29958	5.27E-25
LOC111298611	uncharacterized protein LOC111298611	-6.311471128	0.000469177
MSTRG.33394	Unknown sequences	-6.31791	1.11E-95
LOC111294066	transcription factor CSA-like	-6.318003633	3.40E-39
LOC111283878	uncharacterized LOC111283878	-6.31947	0.000208
MSTRG.9353	Unknown sequences	-6.32033	8.79E-26
MSTRG.16875	Unknown sequences	-6.32179	1.38E-19
MSTRG.29631	Unknown sequences	-6.32458	5.43E-27
MSTRG.24765	Unknown sequences	-6.35591	1.40E-24
LOC111305971	LOB domain-containing protein 1-like	-6.375603275	7.06E-18
LOC111292205	U-box domain-containing protein 27-like	-6.378154499	1.76E-11
LOC111295167	serine/arginine-rich splicing factor SC35-like	-6.38201	8.53E-18
MSTRG.10291	Unknown sequences	-6.40003	7.84E-22
LOC111304854	WD repeat-containing protein 44-like	-6.40018	3.76E-13
LOC111309528	uncharacterized protein LOC111309528	-6.408745624	1.86E-05
LOC111310912	3,9-dihydroxypterocarpan 6A-monooxygenase-like	-6.409674566	8.04E-05
LOC111312349	uncharacterized LOC111312349	-6.41738	1.20E-47
LOC111282412	LOB domain-containing protein 42-like	-6.42836	0.000208
LOC111292392	protein trichome birefringence-like 43	-6.437495032	2.19E-05
LOC111277115	uncharacterized protein LOC111277115	-6.459764	9.03E-57
MSTRG.32718	Unknown sequences	-6.47771	1.60E-41
LOC111289856	traB domain-containing protein isoform X1	-6.495710116	1.50E-29
LOC111316286	probable leucine-rich repeat receptor-like protein kinase At1g35710	-6.49974	1.46E-12
LOC111293859	ubiquitin-conjugating enzyme E2 2	-6.50177	6.27E-15
MSTRG.21056	Unknown sequences	-6.52476	1.93E-13
LOC111295759	zinc finger protein-like 1 homolog	-6.52661	4.08E-107
LOC111305380	alcohol dehydrogenase 1-like	-6.529963658	0.000148747
LOC111313342	myb-related protein 308-like	-6.531272691	6.70E-40
MSTRG.13315	Unknown sequences	-6.53683	1.44E-18
MSTRG.36145	Unknown sequences	-6.54518	3.74E-20
LOC111277239	14 kDa proline-rich protein DC2.15-like	-6.558372723	6.07E-42
LOC111282412	protein=LOB domain-containing protein 42-like	-6.570419784	9.08E-72
LOC111274856	calcyclin-binding protein-like	-6.57107	6.64E-16
LOC111287652	stem-specific protein TSJT1-like	-6.584753109	7.68E-95

Gene Symbol	Gene Name	Log2 fold change	FDR p-value correction
LOC111297851	eukaryotic translation initiation factor 2D	-6.58581	4.00E-154
MSTRG.649	Unknown sequences	-6.60992	7.23E-27
LOC111317381	uncharacterized protein LOC111317381	-6.649742902	4.33E-06
LOC111312151	eukaryotic translation initiation factor 2D	-6.66415	3.83E-12
LOC111284093	putative clathrin assembly protein At1g33340	-6.67899582	4.20E-06
MSTRG.24788	Unknown sequences	-6.72541	3.34E-161
LOC111304242	probable indole-3-pyruvate monooxygenase YUCCA4	-6.73315992	1.99E-05
MSTRG.4788	Unknown sequences	-6.74233	8.39E-51
MSTRG.13935	Unknown sequences	-6.78338	9.04E-46
MSTRG.32418	Unknown sequences	-6.83039	6.56E-22
LOC111309529	putative F-box protein At1g47765	-6.832700784	1.60E-06
LOC111312958	uncharacterized LOC111312958	-6.846234496	1.68E-22
LOC111314256	protein LIFEGUARD 2-like	-6.8786	1.67E-14
LOC111280983	embryo-specific protein ATS3B-like	-6.89428	2.42E-35
LOC111292167	protein SCAI-like	-6.92068	1.92E-22
LOC111287086	probable disease resistance protein At1g58602	-7.0144	1.10E-44
LOC111316151	probable glycosyltransferase At5g03795	-7.0346	1.64E-13
LOC111303853	indole-3-acetaldehyde oxidase-like	-7.151661676	7.02E-07
MSTRG.34919	Unknown sequences	-7.18357	1.71E-16
LOC111276612	LOB domain-containing protein 4-like	-7.243266574	3.36E-07
MSTRG.5465	Unknown sequences	-7.24605	5.07E-28
LOC111318518	uncharacterized LOC111318518	-7.319323386	6.13E-07
MSTRG.27668	Unknown sequences	-7.41324	4.31E-56
LOC111298508	transcription repressor OFP12-like	-7.485007744	1.78E-06
LOC111299024	calmodulin-binding receptor-like cytoplasmic kinase 3	-7.53485	1.50E-20
LOC111296791	uncharacterized LOC111296791	-7.57246	2.46E-21
LOC111290228	leucine-rich repeat receptor protein kinase	-7.574169595	6.34E-09
LOC111311518	uncharacterized LOC111311518	-7.582043405	2.08E-18
LOC111317678	protein SIEVE ELEMENT OCCLUSION B-like	-7.5867	4.24E-05
LOC111274060	probable stress-associated endoplasmic reticulum	-7.600592562	2.30E-08
LOC111289620	probable methyltransferase PMT21	-7.601359298	2.45E-78
LOC111294291	uncharacterized LOC111294291, transcript variant	-7.613542517	2.65E-114
LOC111276730	uncharacterized LOC111276730	-7.6196	0.011557
LOC111314621	putative pentatricopeptide repeat-containing protein At1g19290	-7.63118	0.00268
LOC111274856	calcyclin-binding protein-like	-7.63181	0.003399
LOC111285266	ATP-dependent 6-phosphofructokinase 3-like	-7.6341	0.000101
MSTRG.15958	Unknown sequences	-7.63997	1.13E-05
LOC111281786	probable ubiquitin-conjugating enzyme E2 23	-7.64286	8.40E-06

Gene Symbol	Gene Name	Log2 fold change	FDR p-value correction
MSTRG.32418	Unknown sequences	-7.64693	2.86E-05
LOC111278971	ubiquitin carboxyl-terminal hydrolase 6-like	-7.650372783	8.39E-45
MSTRG.27668	Unknown sequences	-7.65553	1.12E-12
LOC111298394	ferredoxin--nitrite reductase, chloroplastic-like	-7.663576408	6.59E-18
LOC111275121	uncharacterized LOC111275121	-7.67414	0.047556
LOC111318002	bidirectional sugar transporter SWEET10-like	-7.674514843	2.48E-09
LOC111295458	acyl carrier protein 1, chloroplastic-like	-7.68122	0.01566
MSTRG.13583	Unknown sequences	-7.690612198	4.31E-90
MSTRG.2695	Unknown sequences	-7.711985892	4.64E-56
LOC111292167	protein SCAI-like	-7.71438	8.82E-10
LOC111274446	probable disease resistance protein At5g63020	-7.72995	1.00E-05
LOC111311438	transport and Golgi organization 2 homolog	-7.73797	0.0112713
LOC111289620	probable methyltransferase PMT21	-7.75255	1.48E-09
LOC111318703	U-box domain-containing protein 7-like	-7.78136874	2.13E-09
LOC111306326	cysteine-rich repeat secretory protein 60	-7.78927	0.006774
LOC111294291	uncharacterized LOC111294291	-7.81045	0.000208
MSTRG.13583	Unknown sequences	-7.81791	2.88E-10
MSTRG.13316	Unknown sequences	-7.823461033	4.38E-42
LOC111293859	ubiquitin-conjugating enzyme E2 2	-7.82351	5.84E-07
LOC111313563	uncharacterized LOC111313563	-7.841984559	3.24E-10
LOC111304854	WD repeat-containing protein 44-like	-7.85156	0.00259
MSTRG.5465	Unknown sequences	-7.86892	0.000975
MSTRG.2710	Unknown sequences	-7.87387	2.49E-05
MSTRG.5906	Unknown sequences	-7.87783	1.60E-05
LOC111275643	65-kDa microtubule-associated protein 1-like	-7.88051	0.0153
LOC111311769	nucleolar transcription factor 1-B-like,	-7.883230096	5.49E-11
LOC111291655	probable magnesium transporter NIPA6	-7.88339	1.11E-05
LOC111292600	uncharacterized LOC111292600	-7.88564	3.17E-06
LOC111309666	uncharacterized LOC111309666	-7.889628613	8.25E-10
MSTRG.31953	Unknown sequences	-7.92144	4.06E-06
LOC111291600	1,4-alpha-glucan-branching enzyme 1, chloroplastic/amyloplastic-like	-7.93143	1.30E-05
LOC111304214	early nodulin-93-like	-7.957374581	1.07E-109
MSTRG.2695	Unknown sequences	-8.00671	1.44E-11
LOC111274241	transcription factor VOZ1	-8.010812073	3.89E-31
MSTRG.21056	Unknown sequences	-8.04361	0.008704
LOC111278971	ubiquitin carboxyl-terminal hydrolase 6-like	-8.04697	0.000845
MSTRG.5349	Unknown sequences	-8.05037	0.013354
MSTRG.31361	LOC111290245	-8.07112	4.66E-05
LOC111302666	UDP-glycosyltransferase 76E2-like	-8.23364	2.47E-06
MSTRG.13316	Unknown sequences	-8.29971	7.85E-08
MSTRG.14039	Unknown sequences	-8.34373	6.99E-07

Gene Symbol	Gene Name	Log2 fold change	FDR p-value correction
LOC111311552	polygalacturonase inhibitor-like	-8.365194624	1.14E-139
MSTRG.5908	Unknown sequences	-8.37008	1.03E-06
MSTRG.29215	Unknown sequences	-8.41169	0.001064
LOC111311552	polygalacturonase inhibitor-like	-8.47809	0.000208
LOC111287629	small heat shock protein, chloroplastic-like	-8.502309642	6.15E-56
LOC111316686	probable leucine-rich repeat receptor-like protein kinase At1g35710	-8.51171	6.23E-05
LOC111314600	GDP-mannose transporter GONST3-like	-8.55671	1.18E-06
LOC111278395	endoglucanase-like	-8.594383006	4.03E-100
LOC111299987	pentatricopeptide repeat-containing protein At5g39350-like	-8.60895	3.34E-07
LOC111292881	uncharacterized protein At1g04910	-8.71075	0.036341
MSTRG.34919	Unknown sequences	-8.73673	0.005391
LOC111316286	probable leucine-rich repeat receptor-like protein kinase At1g35710	-8.76366	9.38E-08
LOC111311438	transport and Golgi organization 2 homolog	-8.767848142	1.02E-87
LOC111278395	endoglucanase-like	-8.79611	4.36E-13
LOC111287629	small heat shock protein, chloroplastic-like	-8.96268	4.60E-13
LOC111274241	transcription factor VOZ1	-8.99109	4.23E-06
LOC111296791	uncharacterized LOC111296791	-8.99909	6.79E-05
LOC111299024	calmodulin-binding receptor-like cytoplasmic kinase 3	-9.01691	0.000796
LOC111309530	putative F-box protein At3g16210	-9.058104145	6.66E-20
LOC111314256	protein LIFEGUARD 2-like	-9.21108	2.10E-08
LOC111311518	uncharacterized LOC111311518	-9.2998	0.019308
LOC111316151	probable glycosyltransferase At5g03795	-9.3103	0.01446
LOC111282902	uncharacterized LOC111282902	-9.697481644	1.92E-26
LOC111304216	early nodulin-93-like	-11.0197445	9.60E-139
