# Supplementary material for: Transcriptome analysis during fruit developmental stages in durian (Durio zibethinus Murr.) var. D24
Source: Genet Mol Biol. 2023 Jan 6;45(4):e20210379. doi: 10.1590/1678-4685-GMB-2021-0379 (PMC9830936; doi:10.1590/1678-4685-GMB-2021-0379)
Supplement: Table S5 - [file 1415-4757-GMB-45-4-e20210379-s7.pdf]

Genetics and Molecular Biology
DOI: https://doi.org/10.1590/1678-4685-GMB-2021-0379

Supplementary Material to "Transcriptome analysis during fruit developmental stages in durian (*Durio zibethinus* Murr.) var. D24"

Table S5 - Categorisation of expressed genes to Gene Ontology terms in YS/MS, YS/RS, and MS/RS.

a. Biological Process [P]

Level	GO ID	GO Name [Biological Process]	YS/MS	YS/RS	MS/RS
1	GO:0008150	biological process	1870	1149	2038
2	GO:0008152	metabolic process	1416	862	1509
2	GO:0009987	cellular process	1401	806	1415
2	GO:0050896	response to stimulus	395	227	393
2	GO:0071840	cellular component organization or biogenesis	228	139	219
2	GO:0032502	developmental process	210	0	0
2	GO:0051179	localization	0	116	208
3	GO:0044237	cellular metabolic process	1204	700	1169
3	GO:0006807	nitrogen compound metabolic process	1075	620	1084
3	GO:0071704	organic substance metabolic process	1042	520	1009
3	GO:0044238	primary metabolic process	1042	520	1009
3	GO:0009058	biosynthetic process	674	346	652
3	GO:0044281	small molecule metabolic process	432	245	471
3	GO:0006950	response to stress	277	174	291
3	GO:0016043	cellular component organization	215	128	0
3	GO:0048856	anatomical structure development	210	0	0
3	GO:0009056	catabolic process	0	161	209
3	GO:0051234	establishment of localization	0	116	208
4	GO:0043170	macromolecule metabolic process	673	333	555
4	GO:0044260	cellular macromolecule metabolic process	621	306	534
4	GO:1901564	organonitrogen compound metabolic process	610	308	573
4	GO:0034641	cellular nitrogen compound metabolic process	608	374	639
4	GO:0019538	protein metabolic process	541	273	484

Level	GO ID	GO Name [Biological Process]	YS/MS	YS/RS	MS/RS
4	GO:0006082	organic acid metabolic process	248	0	205
4	GO:0006520	cellular amino acid metabolic process	248	0	205
4	GO:0006629	lipid metabolic process	196	120	231
4	GO:0006810	transport	0	116	208
5	GO:0044267	cellular protein metabolic process	533	271	480
5	GO:0043412	macromolecule modification	490	246	444
5	GO:0036211	protein modification process	490	246	0
5	GO:0043436	oxoacid metabolic process	248	0	205
6	GO:0006464	cellular protein modification process	490	246	444
6	GO:0019752	carboxylic acid metabolic process	248	0	205

b. Molecular Function [F]

Level	GO ID	GO Name [molecular_function]	YS/MS	YS/RS	MS/RS
1	GO:0003674	molecular function	2876	1646	3160
2	GO:0005488	binding	1699	894	1601
2	GO:0003824	catalytic activity	1427	730	1571
3	GO:0043167	ion binding	1137	597	1094
3	GO:0016740	transferase activity	764	0	649
3	GO:0097159	organic cyclic compound binding	631	335	504
3	GO:1901363	heterocyclic compound binding	631	335	504
3	GO:0016787	hydrolase activity	323	166	519
3	GO:0016740	transferase activity	0	247	0
4	GO:0003676	nucleic acid binding	631	335	504
4	GO:0016772	transferase activity, transferring phosphorus-containing groups	459	0	358
5	GO:0003723	RNA binding	359	0	0
5	GO:0016301	kinase activity	354	0	329
5	GO:0003677	DNA binding	301	196	344

c. Cellular Component [C]

Level	GO ID	GO Name [cellular_component]	YS/MS	YS/RS	MS/RS
1	GO:0005575	cellular function	1989	1103	2112
2	GO:0005623	cell	1297	775	1354
2	GO:0044464	cell part	1273	746	1269
2	GO:0043226	organelle	1049	533	969

Level	GO ID	GO Name [cellular_component]	YS/MS	YS/RS	MS/RS
2	GO:0032991	protein-containing complex	388	258	595
3	GO:0005622	intracellular	1208	709	1220
3	GO:0044424	intracellular part	1166	653	1114
3	GO:0043229	intracellular organelle	1042	527	952
3	GO:0043227	membrane-bounded organelle	951	454	796
3	GO:0043228	non-membrane-bounded organelle	0	149	256
4	GO:0043231	intracellular membrane-bounded organelle	911	452	757
4	GO:0005737	cytoplasm	690	395	663
4	GO:0044444	cytoplasmic part	558	270	508
4	GO:0043232	intracellular non-membrane-bounded organelle	0	149	256
5	GO:0005634	nucleus	563	308	504
