# Supplementary material for: Transcriptome analysis during fruit developmental stages in durian (Durio zibethinus Murr.) var. D24
Source: Genet Mol Biol. 2023 Jan 6;45(4):e20210379. doi: 10.1590/1678-4685-GMB-2021-0379 (PMC9830936; doi:10.1590/1678-4685-GMB-2021-0379)
Supplement: Table S6 - [file 1415-4757-GMB-45-4-e20210379-s8.pdf]

Genetics and Molecular Biology
DOI: https://doi.org/10.1590/1678-4685-GMB-2021-0379

Supplementary Material to "Transcriptome analysis during fruit developmental stages in durian (*Durio zibethinus* Murr.) var. D24"

Table S6 - KEGG pathway term distribution of expressed genes in YS/MS, YS/RS, and MS/RS.

KEGG pathway terms	Map	YS/MS	YS/RS	MS/RS
Nucleotide Metabolism				
Purine metabolism	map00230	138	68	185
Pyrimidine metabolism	map00240	4	7	7
Metabolism of cofactors and vitamins				
Thiamine metabolism	map00730	138	70	180
Nicotinate and nicotinamide metabolism	map00760	18	6	5
Ubiquinone and other terpenoid-quinone biosynthesis	map00130	16	13	2
One carbon pool by folate	map00670	6	0	1
Pantothenate and CoA biosynthesis	map00770	5	3	1
Riboflavin metabolism	map00740	5	2	1
Porphyrin and chlorophyll metabolism	map00860	4	7	3
Vitamin B6 metabolism	map00750	3	0	1
Folate biosynthesis	map00790	1	4	2
Biotin metabolism	map00780	0	1	1
Lipoic acid metabolism	map00785	0	0	1
Retinol metabolism	map00830	4	3	2
Carbohydrate metabolism				
**Starch and sucrose metabolism**	**map00500**	**85**	**8**	**15**
Amino sugar and nucleotide sugar metabolism	map00520	57	19	15
Glycolysis / Gluconeogenesis	map00010	24	15	11
Galactose metabolism	map00052	16	15	11
Pentose and glucuronate interconversions	map00040	14	4	4
Pentose phosphate pathway	map00030	13	1	2
Inositol phosphate metabolism	map00562	9	3	1
Glyoxylate and dicarboxylate metabolism	map00630	8	3	2
Fructose and mannose metabolism	map00051	7	6	3
Citrate cycle (TCA cycle)	map00020	4	11	4

KEGG pathway terms	Map	YS/MS	YS/RS	MS/RS
Butanoate metabolism	map00650	2	5	4
Ascorbate and aldarate metabolism	map00053	0	2	5
C5-Branched dibasic acid metabolism	map00660	0	0	1
Pyruvate metabolism	map00620	15	10	7
Genetic Information Processing				
Aminoacyl-tRNA biosynthesis	map00970	26	7	3
Lipid Metabolism				
Glycerophospholipid metabolism	map00564	25	24	9
Glycerolipid metabolism	map00561	16	14	14
Ether lipid metabolism	map00565	14	15	6
Sphingolipid metabolism	map00600	9	1	2
Arachidonic acid metabolism	map00590	5	10	2
Fatty acid biosynthesis	map00061	3	0	4
Synthesis and degradation of ketone bodies	map00072	2	2	2
Fatty acid degradation	map00071	1	2	3
Linoleic acid metabolism	map00591	1	0	0
alpha-Linolenic acid metabolism	map00592	0	5	6
Cutin, suberine and wax biosynthesis	map00073	0	0	7
Linoleic acid metabolism	map00591	0	4	2
Steroid biosynthesis	map00100	0	0	2
Steroid hormone biosynthesis	map00140	3	0	2
Biosynthesis of other Secondary Metabolites				
Phenylpropanoid biosynthesis	map00940	25	9	5
Caffeine metabolism	map00232	14	12	2
Isoquinoline alkaloid biosynthesis	map00950	9	0	3
Tropane, piperidine and pyridine alkaloid biosynthesis	map00960	9	1	2
Glucosinolate biosynthesis	map00966	4	3	0
Flavonoid biosynthesis	map00941	1	0	0
Biosynthesis of secondary metabolites - unclassified	map00999	0	0	1
Indole alkaloid biosynthesis	map00901	1	1	1
Novobiocin biosynthesis	map00401	9	0	0
Neomycin, kanamycin and gentamicin biosynthesis	map00524	5	4	1
Streptomycin biosynthesis	map00521	5	4	1
Phenazine biosynthesis	map00405	0	0	1
Amino Acid Metabolism				
Cysteine and methionine metabolism	map00270	20	11	9

KEGG pathway terms	Map	YS/MS	YS/RS	MS/RS
Arginine and proline metabolism	map00330	18	7	4
Glycine, serine and threonine metabolism	map00260	14	6	4
Valine, leucine and isoleucine biosynthesis	map00290	13	6	2
Alanine, aspartate and glutamate metabolism	map00250	12	6	4
Arginine biosynthesis	map00220	12	4	2
Tyrosine metabolism	map00350	12	2	11
Phenylalanine metabolism	map00360	10	1	0
Phenylalanine, tyrosine and tryptophan biosynthesis	map00400	9	2	5
Valine, leucine and isoleucine degradation	map00280	8	5	2
Histidine metabolism	map00340	3	1	2
Tryptophan metabolism	map00380	3	1	2
Lysine biosynthesis	map00300	2	2	0
Phenylalanine metabolism	map00360	0	0	4
Methane metabolism	map00680	7	6	5
Lysine degradation	map00310	33	5	3
Environmental Information Processing				
Phosphatidylinositol signaling system	map04070	19	9	2
Energy Metabolism				
Carbon fixation in photosynthetic organisms	map00710	18	9	3
Oxidative phosphorylation	map00190	10	0	6
**Sulphur metabolism**	**map00920**	**6**	**2**	**13**
Biosynthesis of unsaturated fatty acids	map01040	1	0	1
**Nitrogen metabolism**	**map00910**	**1**	**16**	**2**
Carbon fixation pathways in prokaryotes	map00720	1	8	2
Metabolism of other amino acids				
Cyanoamino acid metabolism	map00460	6	0	2
**Glutathione metabolism**	**map00480**	**5**	**18**	**5**
beta-Alanine metabolism	map00410	2	4	5
Selenocompound metabolism	map00450	1	1	3
Phosphonate and phosphinate metabolism	map00440	0	0	1
Taurine and hypotaurine metabolism	map00430	0	3	1
D-Glutamine and D-glutamate metabolism	map00471	1	1	2
Glycan biosynthesis and metabolism				
Glycosphingolipid biosynthesis - globo and isoglobo series	map00603	6	1	0
Glycosylphosphatidylinositol (GPI)-anchor biosynthesis	map00563	5	0	0

KEGG pathway terms	Map	YS/MS	YS/RS	MS/RS
Glycosphingolipid biosynthesis - ganglio series	map00604	4	0	1
Other glycan degradation	map00511	3	0	2
Glycosaminoglycan degradation	map00531	2	0	1
Glycosphingolipid biosynthesis - lacto and neolacto series	map00601	2	0	0
N-Glycan biosynthesis	map00510	0	0	1
Mucin type O-glycan biosynthesis	map00512	2	0	0
Various types of N-glycan biosynthesis	map00513	2	0	0
Lipopolysaccharide biosynthesis	map00540	2	1	0
Glycosaminoglycan biosynthesis - heparan sulfate / heparin	map00534	2	2	0
Glycosaminoglycan biosynthesis - keratan sulfate	map00533	2	0	0
Lipopolysaccharide biosynthesis	map00540	0	0	2
Peptidoglycan biosynthesis	map00550	0	0	1
Metabolism of terpenoids and polyketides				
Diterpenoid biosynthesis	map00904	3	0	0
Carotenoid biosynthesis	map00906	1	0	1
Terpenoid backbone biosynthesis	map00900	2	0	5
Limonene and pinene degradation	map00903	0	0	1
Sesquiterpenoid and triterpenoid biosynthesis	map00909	0	0	1
Geraniol degradation	map00281	2	2	0
Insect hormone biosynthesis	map00981	0	0	1
Global Pathway				
Biosynthesis of antibiotics	map01130	77	41	33
Aminobenzoate degradation	map00627	1	2	0
Naphthalene degradation	map00626	1	0	1
Steroid degradation	map00984	1	0	2
Drug metabolism - cytochrome P450	map00982	11	7	4
Drug metabolism - other enzymes	map00983	29	24	7
Nitrotoluene degradation	map00633	13	11	1
Metabolism of xenobiotics by cytochrome P450	map00980	5	7	3
Styrene degradation	map00643	0	0	5
Caprolactam degradation	map00930	0	2	1
Chloroalkane and chloroalkene degradation	map00625	0	1	2
Environmental Information Processing; Signal transduction				
mTOR signaling pathway	map04150	1	0	1

KEGG pathway terms	Map	YS/MS	YS/RS	MS/RS
Immune System				
Th1 and Th2 cell differentiation	map04658	33	24	1
T cell receptor signaling pathway	map04660	37	24	2
