# Supplementary material for: Transcriptome analysis during fruit developmental stages in durian (Durio zibethinus Murr.) var. D24
Source: Genet Mol Biol. 2023 Jan 6;45(4):e20210379. doi: 10.1590/1678-4685-GMB-2021-0379 (PMC9830936; doi:10.1590/1678-4685-GMB-2021-0379)
Supplement: Table S7 - [file 1415-4757-GMB-45-4-e20210379-s9.pdf]

Genetics and Molecular Biology
DOI: https://doi.org/10.1590/1678-4685-GMB-2021-0379

Supplementary Material to "Transcriptome analysis during fruit developmental stages in durian (*Durio zibethinus* Murr.) var. D24"

Table S7 - Heatmap results for softening genes.

Clust_#	Log2 fold change (Transition from mature stage to ripening stage)	Log2 fold change (Transition from young stage to mature stage)	Gene Symbol	Gene Name
Cluster_3	2.35818	-3.38712	LOC111291927	cellulose synthase-like protein G2
Cluster_3	2.40258	-3.57443	LOC111286780	beta-galactosidase 8, X1
Cluster_3	2.63924	-3.47159	LOC111311338	U-box domain-containing protein 4-like
Cluster_3	2.76578	-3.7092	LOC111315475	probable xyloglucan glycosyltransferase 12
Cluster_3	2.73849	-3.7626	LOC111276893	cellulose synthase-like protein D3,
Cluster_3	2.09965	-4.40848	LOC111277096	U-box domain-containing protein 4-like
Cluster_3	1.97062	-4.34637	LOC111293168	cellulose synthase A catalytic subunit 2
Cluster_3	1.97521	-4.08394	LOC111296013	cellulose synthase-like protein D3,
Cluster_3	3.36652	-4.06387	LOC111291010	expansin-A15-like
Cluster_3	2.98611	-2.64462	LOC111296009	beta-glucosidase BoGH3B-like
Cluster_3	3.39961	-2.53281	LOC111274640	beta-galactosidase 17-like,
Cluster_3	2.90944	-5.89373	LOC111303421	probable xyloglucan

Clust_#	Log2 fold change (Transition from mature stage to ripening stage)	Log2 fold change (Transition from young stage to mature stage)	Gene Symbol	Gene Name
Cluster_3	2.98039	-5.86174	LOC111286758	beta-galactosidase-like
Cluster_3	3.44781	-6.57845	LOC111289825	pectin acetylesterase 7-like,
Cluster_3	3.89605	-5.41078	LOC111303852	beta-glucosidase BoGH3B-like
Cluster_3	2.7772	-4.96765	LOC111284692	xyloglucan endotransglucosylase/hydrolase
Cluster_3	2.79502	-5.2204	LOC111304738	U-box domain-containing protein 33-like,
Cluster_3	2.43431	-5.2358	LOC111305459	U-box domain-containing protein 9-like
Cluster_3	5.19339	-6.32033	LOC111291944	xyloglucan glycosyltransferase 4-like
Cluster_3	5.25215	-6.70076	LOC111285714	xyloglucan endotransglucosylase/hydrolase
Cluster_3	5.46311	-5.63575	LOC111274750	pectinesterase-like
Cluster_3	6.05873	-7.36103	LOC111287953	xyloglucan endotransglucosylase/hydrolase
Cluster_3	6.1364	-7.3922	LOC111292968	probable xyloglucan
Cluster_3	3.94808	-8.02948	LOC111303423	probable xyloglucan
Cluster_3	3.62892	-8.29518	LOC111274745	pectinesterase 3
Cluster_3	9.03817	-11.0334	LOC111278395	endoglucanase-like
Cluster_2	0	-3.28599	LOC111318030	U-box domain-containing protein 11-like,
Cluster_2	0	-3.23435	LOC111312181	cellulose synthase A catalytic subunit 5
Cluster_2	0	-3.33978	LOC111304673	xyloglucan endotransglycosylase/hydrolase
Cluster_2	0	-3.31337	LOC111296397	polygalacturonase inhibitor 1

Clust_#	Log2 fold change (Transition from mature stage to ripening stage)	Log2 fold change (Transition from young stage to mature stage)	Gene Symbol	Gene Name
Cluster_2	0	-3.18168	LOC111280747	U-box domain-containing protein 4-like
Cluster_2	0	-3.18452	LOC111274630	beta-galactosidase 1
Cluster_2	0	-3.14884	LOC111311190	U-box domain-containing protein 29-like
Cluster_2	0	-3.02487	LOC111317984	U-box domain-containing protein 6-like,
Cluster_2	0	-2.99743	LOC111279254	probable xyloglucan glycosyltransferase 12,
Cluster_2	0	-2.91779	LOC111298977	cellulose synthase A catalytic subunit 1
Cluster_2	0	-2.78119	LOC111278693	U-box domain-containing protein 4-like
Cluster_2	0	-2.74939	LOC111309977	beta-galactosidase, X1
Cluster_2	0	-2.61443	LOC111306340	U-box domain-containing protein 21
Cluster_2	0	-2.59038	LOC111315722	cellulose synthase A catalytic subunit 5
Cluster_2	0	-3.57817	LOC111315695	U-box domain-containing protein 29-like
Cluster_2	0	-3.52013	LOC111288407	probable xyloglucan
Cluster_2	0	-3.46012	LOC111312526	probable xyloglucan glycosyltransferase 12
Cluster_2	0	-3.78156	LOC111312616	probable xyloglucan 6-xylosyltransferase 5
Cluster_2	0	-3.77137	LOC111316883	beta-galactosidase 10-like

Clust_#	Log2 fold change (Transition from mature stage to ripening stage)	Log2 fold change (Transition from young stage to mature stage)	Gene Symbol	Gene Name
Cluster_2	0	-3.69578	LOC111289968	U-box domain-containing protein 33-like,
Cluster_2	0	-4.02578	LOC111289648	expansin-like B1, X1
Cluster_2	0	-3.98731	LOC111275321	endoglucanase 25-like
Cluster_2	0	-3.89443	LOC111303111	beta-galactosidase-like
Cluster_2	0	-4.18466	LOC111275267	U-box domain-containing protein 4-like,
Cluster_2	0	-4.0887	LOC111288463	U-box domain-containing protein 18
Cluster_2	0	-1.71824	LOC111317186	U-box domain-containing protein 5-like,
Cluster_2	0	-1.71082	LOC111278000	U-box domain-containing protein 30-like
Cluster_2	0	-1.68645	LOC111317549	U-box domain-containing protein 21-like
Cluster_2	0	-1.78624	LOC111295948	U-box domain-containing protein 14-like
Cluster_2	0	-1.78937	LOC111280838	beta-galactosidase 17-like,
Cluster_2	0	-1.76237	LOC111318270	beta-glucosidase 40-like, X1
Cluster_2	0	-1.52835	LOC111313678	cellulose synthase A catalytic subunit 3
Cluster_2	0	-2.04035	LOC111304739	U-box domain-containing protein 33-like,
Cluster_2	0	-2.06938	LOC111283356	probable xyloglucan glycosyltransferase 6,
Cluster_2	0	-2.11865	LOC111314351	xyloglucan galactosyltransferase MUR3-like

Clust_#	Log2 fold change (Transition from mature stage to ripening stage)	Log2 fold change (Transition from young stage to mature stage)	Gene Symbol	Gene Name
Cluster_2	0	-2.10863	LOC111286079	pectinesterase/pectinesterase inhibitor
Cluster_2	0	-2.0059	LOC111313303	xyloglucan endotransglycosylase/hydrolase
Cluster_2	0	-2.0191	LOC111276820	beta-glucosidase 42-like, X1
Cluster_2	0	-1.9904	LOC111277138	pectin acetylesterase 12-like,
Cluster_2	-1.54482	-1.81964	LOC111284741	cellulose synthase A catalytic subunit 1
Cluster_2	-3.37767	-1.92762	LOC111278980	beta-galactosidase 1-like
Cluster_2	0	-6.98037	LOC111283944	xyloglucan galactosyltransferase MUR3-like
Cluster_2	0	-6.74319	LOC111291258	U-box domain-containing protein 19-like
Cluster_2	0	-6.42496	LOC111291886	beta-galactosidase 3-like
Cluster_2	0	-7.32199	LOC111299213	cellulose synthase A catalytic subunit 5
Cluster_2	0	-5.11718	LOC111298783	probable xyloglucan galactosyltransferase GT17
Cluster_2	0	-5.08399	LOC111298117	expansin-like A2
Cluster_2	0	-5.03538	LOC111281000	endoglucanase 25-like
Cluster_2	0	-4.799	LOC111304491	cellulose synthase A catalytic subunit 3
Cluster_2	0	-4.73241	LOC111299804	cellulose synthase A catalytic subunit 2
Cluster_2	0	-5.58779	LOC111307823	xyloglucan endotransglucosylase/hydrolase

Clust_#	Log2 fold change (Transition from mature stage to ripening stage)	Log2 fold change (Transition from young stage to mature stage)	Gene Symbol	Gene Name
Cluster_2	0	-5.41067	LOC111304740	U-box domain-containing protein 33-like
Cluster_1	0	3.25174	LOC111299778	U-box domain-containing protein 27-like
Cluster_1	0	3.18304	LOC111282609	probable xyloglucan
Cluster_1	0	3.41219	LOC111318703	U-box domain-containing protein 7-like
Cluster_1	0	3.7957	LOC111311521	polygalacturonase-like
Cluster_1	0	2.45962	LOC111314441	U-box domain-containing protein 9-like
Cluster_1	0	2.47978	LOC111290971	expansin-like B1
Cluster_1	0	2.29027	LOC111294605	probable xyloglucan glycosyltransferase 9,
Cluster_1	0	2.09218	LOC111281864	putative U-box domain-containing protein 42,
Cluster_1	-1.56103	4.61035	LOC111285245	probable xyloglucan galactosyltransferase GT11,
Cluster_1	-3.12501	2.02888	LOC111317423	pectinesterase 3-like
Cluster_1	0	8.43397	LOC111311552	polygalacturonase inhibitor-like
