# Supplementary material for: Transcriptome analysis during fruit developmental stages in durian (Durio zibethinus Murr.) var. D24
Source: Genet Mol Biol. 2023 Jan 6;45(4):e20210379. doi: 10.1590/1678-4685-GMB-2021-0379 (PMC9830936; doi:10.1590/1678-4685-GMB-2021-0379)
Supplement: Table S8 - [file 1415-4757-GMB-45-4-e20210379-s10.pdf]

Genetics and Molecular Biology
DOI: https://doi.org/10.1590/1678-4685-GMB-2021-0379

Supplementary Material to "Transcriptome analysis during fruit developmental stages in durian (*Durio zibethinus* Murr.) var. D24"

Table S8 - Heatmap results for signal transduction.

1. Genes involved in ethylene biosynthesis.

Gene Symbol	Gene Name	Log2 fold change (Transition from young stage to mature stage)	Log2 fold change (Transition from mature stage to ripening stage)
LOC111314546	1-aminocyclopropane-1-carboxylate oxidase	-5.10515	4.57221
LOC111296866	1-aminocyclopropane-1-carboxylate oxidase	-5.42301	3.30056
LOC111317524	1-aminocyclopropane-1-carboxylate oxidase	-2.24663	2.43102
LOC111306707	1-aminocyclopropane-1-carboxylate oxidase	2.41753	0
LOC111305273	1-aminocyclopropane-1-carboxylate oxidase	-3.95841	0
LOC111274040	1-aminocyclopropane-1-carboxylate oxidase-like	-4.43793	3.24625
LOC111300706	1-aminocyclopropane-1-carboxylate synthase	-2.09305	-3.28987
LOC111313897	1-aminocyclopropane-1-carboxylate synthase-like	-2.80886	1.94419
LOC111304783	1-aminocyclopropane-1-carboxylate synthase-like	1.61627	0
LOC111299814	S-adenosylmethionine carrier 1	-2.27598	0
LOC111286226	S-adenosylmethionine decarboxylase	-2.96624	5.16461
LOC111287069	S-adenosylmethionine decarboxylase	-2.65215	3.66747
LOC111318202	S-adenosylmethionine decarboxylase	-2.65431	2.18832
LOC111282309	S-adenosylmethionine decarboxylase	-1.74857	1.8788
LOC111304250	S-adenosylmethionine decarboxylase	-4.8777	0
LOC111304951	S-adenosylmethionine synthase 2	-1.72133	-1.5831
LOC111279193	S-adenosylmethionine synthase 2	-5.6817	2.0695

2. Genes involved in ethylene signalling pathway.

Gene Symbol	Gene Name	Log2 fold change (Transition from young stage to mature stage)	Log2 fold change (Transition from mature stage to ripening stage)
LOC111284689	EIN3-binding F-box protein 1-like	1.96413	-1.74249
LOC111303272	EIN3-binding F-box protein 1-like	2.7235	0
LOC111279153	ethylene receptor 2-like	3.36653	0
LOC111306700	ethylene receptor 2-like	2.8125	-2.07699
LOC111291911	ethylene receptor 2-like	2.05944	-2.76811
LOC111305038	ethylene response sensor 1-like	-1.73411	0
LOC111295068	ethylene-overproduction protein 1-like	0	1.82714
LOC111286414	ethylene-overproduction protein 1-like	0	-1.80587
LOC111282084	mitogen-activated protein kinase 17-like	-1.65344	3.15983
LOC111275978	mitogen-activated protein kinase 19-like	-1.95352	0
LOC111284718	mitogen-activated protein kinase 3	-2.2638	2.62908
LOC111282030	mitogen-activated protein kinase 9-like	-1.67659	0
LOC111289681	mitogen-activated protein kinase homolog	2.62611	0
LOC111301438	mitogen-activated protein kinase homolog NTF6	-2.62893	0
LOC111275659	mitogen-activated protein kinase kinase kinase	-8.08745	4.69845
LOC111306687	mitogen-activated protein kinase kinase kinase	-6.06221	3.81307
LOC111290499	mitogen-activated protein kinase kinase kinase	-2.24909	1.81575
LOC111315610	mitogen-activated protein kinase kinase kinase	-2.04864	1.60796
LOC111285643	mitogen-activated protein kinase kinase kinase	2.69438	0
LOC111315404	mitogen-activated protein kinase kinase kinase	-2.69059	0
LOC111292037	mitogen-activated protein kinase kinase kinase	-3.94503	0
LOC111301871	mitogen-activated protein kinase kinase kinase	-4.09542	0
LOC111318818	mitogen-activated protein kinase kinase kinase	-7.37864	0
LOC111312465	serine/threonine-protein kinase CTR1-like	-2.18159	2.47455

3. Genes involved in ethylene response.

Gene Symbol	Gene Name	Log2 fold change (Transition from young stage to mature stage)	Log2 fold change (Transition from mature stage to ripening stage)
LOC111290879	ethylene-responsive transcription factor	-3.9508	3.77611
LOC111304158	ethylene-responsive transcription factor	-4.23178	3.28427
LOC111280307	ethylene-responsive transcription factor	-4.65325	3.01434
LOC111301172	ethylene-responsive transcription factor	-2.91794	2.85424
LOC111292151	ethylene-responsive transcription factor	-3.99649	2.64843
LOC111292116	ethylene-responsive transcription factor	-2.92381	2.64577
LOC111296272	ethylene-responsive transcription factor	-5.15796	2.63686
LOC111304157	ethylene-responsive transcription factor	-2.9678	2.0609
LOC111299257	ethylene-responsive transcription factor	-5.07856	1.59693
LOC111306880	ethylene-responsive transcription factor	4.59281	0
LOC111314102	ethylene-responsive transcription factor	2.58359	0
LOC111306123	ethylene-responsive transcription factor	1.93404	0
LOC111299205	ethylene-responsive transcription factor	1.69509	0
LOC111285490	ethylene-responsive transcription factor	-1.82564	0
LOC111279035	ethylene-responsive transcription factor	-1.85751	0
LOC111287270	ethylene-responsive transcription factor	-1.9127	0
LOC111318106	ethylene-responsive transcription factor	-2.21131	0
LOC111295193	ethylene-responsive transcription factor	-2.38553	0
LOC111279759	ethylene-responsive transcription factor	-2.45319	0
LOC111299848	ethylene-responsive transcription factor	-2.52998	0
LOC111300672	ethylene-responsive transcription factor	-2.83074	0
LOC111312376	ethylene-responsive transcription factor	-2.83595	0
LOC111311178	ethylene-responsive transcription factor	-3.07038	0

LOC111309917	ethylene-responsive transcription factor	-3.4791	0
LOC111305283	ethylene-responsive transcription factor	-3.66669	0
LOC111309281	ethylene-responsive transcription factor	-3.80244	0
LOC111308283	ethylene-responsive transcription factor	-3.85505	0
LOC111293640	ethylene-responsive transcription factor	-4.00151	0
LOC111311243	ethylene-responsive transcription factor	-4.20813	0
LOC111316955	ethylene-responsive transcription factor	-4.80753	0
LOC111300396	ethylene-responsive transcription factor	-4.84218	0
LOC111309418	ethylene-responsive transcription factor	-5.50105	0
LOC111315585	ethylene-responsive transcription factor	-5.68808	0
LOC111315804	ethylene-responsive transcription factor	-7.35398	0
LOC111285645	ethylene-responsive transcription factor	1.59995	-1.5414
LOC111304137	ethylene-responsive transcription factor	-2.61087	-2.96681
LOC111299480	ethylene-responsive transcription factor	3.77712	-3.3807
LOC111307395	ethylene-responsive transcription factor 1B	-3.30071	0
LOC111276899	ethylene-responsive transcription factor 2-like	-1.86055	2.74019
LOC111291204	ethylene-responsive transcription factor 2-like	-3.92165	2.13609
LOC111290760	ethylene-responsive transcription factor 2-like	-2.68887	0
LOC111276522	ethylene-responsive transcription factor 4-like	-2.1634	0
LOC111278637	ethylene-responsive transcription factor 4-like	-4.33223	0
LOC111275030	ethylene-responsive transcription factor 4-like	-4.74314	0
LOC111291396	ethylene-responsive transcription factor ERF061	-7.03836	5.12252
LOC111290737	ethylene-responsive transcription factor ERF113	1.57019	0
