# Supplementary material for: Transcriptome analysis during fruit developmental stages in durian (Durio zibethinus Murr.) var. D24
Source: Genet Mol Biol. 2023 Jan 6;45(4):e20210379. doi: 10.1590/1678-4685-GMB-2021-0379 (PMC9830936; doi:10.1590/1678-4685-GMB-2021-0379)
Supplement: Table S9 - [file 1415-4757-GMB-45-4-e20210379-s11.pdf]

Genetics and Molecular Biology
DOI: https://doi.org/10.1590/1678-4685-GMB-2021-0379

Supplementary Material to "Transcriptome analysis during fruit developmental stages in durian (*Durio zibethinus* Murr.) var. D24"

Table S9 - Genes and metabolizing enzymes involved in starch and sucrose metabolism pathway.

1. Identified genes in the starch and sucrose metabolism pathway.

a. Transition from young to mature stage

Locus (Chr:Start-End)	Metabolizing enzymes	Gene symbol	Description
NW_019167937.1:31150242-31160456	EC:5.3.1.9 - isomerase	LOC111312441	*uncharacterized LOC111312441*
NW_019167960.1:7870010-7870999	EC:2.4.1.34 - synthase	LOC111316836	*uncharacterized LOC111316836*
NW_019168015.1:14014159-14021413	EC:2.4.1.34 - synthase	LOC111278181	callose synthase 11-like
NW_019167938.1:15505567-15524846	EC:2.4.1.18 - branching enzyme	LOC111314672	1,4-alpha-glucan-branching enzyme 2-1,chloroplastic/amyloplastic-like
NW_019168426.1:6282-21213	EC:2.4.1.18 - branching enzyme	LOC111291600	1,4-alpha-glucan-branching enzyme 1,chloroplastic/amyloplastic-like
NW_019167960.1:9302267-9305462	EC:2.7.7.27 - adenylyltransferase	LOC111316856	glucose-1-phosphate adenylyltransferase large subunit 1, chloroplastic-like
NW_019168481.1:25094521-25097000	EC:2.7.1.4 - fructokinase (phosphorylating)	LOC111293926	fructokinase-like 1, chloroplastic
NW_019167882.1:22167487-22174212	EC:2.7.1.1 - hexokinase type IV glucokinase	LOC111303941	hexokinase-3-like

b. Transition from young to ripening stage

Locus (Chr:Start-End)	Metabolizing enzymes	Gene symbol	Description
NW_019167849.1:10546605-10554032	EC:2.4.1.34 - synthase	LOC1112911135	callose synthase 12-like
NW_019167882.1:22167487-22174212	EC:2.7.1.1 - hexokinase type IV glucokinase	LOC111303941	hexokinase-3-like
NW_019168481.1:21661427-21668986	EC:2.4.1.12 - synthase (UDP-forming)	LOC111293564	probable cellulose synthase A catalytic subunit 8 [UDP-forming]
NW_019168481.1:8521803-8523746	EC:2.4.1.12 - synthase (UDP-forming)	LOC111293942	4-alpha-glucanotransferase DPE2

c. Transition from mature to ripening stage

Locus (Chr:Start-End)	Metabolizing enzymes	Gene symbol	Description
NW_019167937.1:31150242-31160456	EC:3.2.1.39 - endo-1,3-beta-D-glucosidase	LOC111312441	*uncharacterized LOC111312441*
NW_019168015.1:14014159-14021413	EC:2.4.1.34 - synthase	LOC111278181	callose synthase 11-like
NW_019167960.1:9302267-9305462	EC:2.7.7.27 - adenylyltransferase	LOC111316856	glucose-1-phosphate adenylyltransferase large subunit 1, chloroplastic-like
NW_019167882.1:22167487-22174212	EC:2.7.1.1 - hexokinase type IV glucokinase	LOC111303941	hexokinase-3-like
NW_019168481.1:25094521-25097000	EC:2.7.1.4 - fructokinase (phosphorylating)	LOC111293926	fructokinase-like 1, chloroplastic
NW_019167960.1:7870010-7870999	EC:2.7.7.27 - adenylyltransferase	LOC111316836	*uncharacterized LOC111316836*
NW_019167937.1:2,192,345-2,196,476	EC:3.1.3.24 - phosphatase	LOC111311629	sucrose-phosphatase 2-like
NW_019168159.1:25,486,523-25,495,125	EC:2.4.1.13 - synthase	LOC111286073	probable sucrose-phosphate synthase 1
NW_019167937.1:33,919,794-33,922,417	EC:3.2.1.39 - endo-1,3-beta-D-glucosidase	LOC111312703	glucan endo-1,3-beta-D-glucosidase-like
NW_019167838.1:4,158,516-4,160,995	EC:3.2.1.4 - endo-1,4-beta-D-glucanase	LOC111278395	endoglucanase-like
NW_019167838.1:6,	EC:2.4.1.12 -	LOC111287763	probable trehalose-phosphate

756,338-6,763,193	synthase (UDP-forming)		phosphatase D
NW_019168381.1:26,223,140-26,228,064	EC:2.4.1.12 - synthase (UDP-forming)	LOC111290775	acid beta-fructofuranosidase 1, vacuolar-like
NW_019167904.1:18,187,419-18,190,194	EC:3.2.1.39 - endo-1,3-beta-D-glucosidase	LOC111306779	beta-amylase 1, chloroplastic-like
NW_019168026.1:2,088,092-2,091,258	EC:3.2.1.2 - saccharogen amylase	LOC111279622	beta-amylase 1, chloroplastic-like
NW_019167904.1:20,121,034-20,122,845	EC:3.2.1.39 - endo-1,3-beta-D-glucosidase	LOC111306922	glucan endo-1,3-beta-glucosidase, basic vacuolar isoform
NW_019167937.1_33173237_33175372_plus	EC:3.2.1.1 - glycogenase	LOC111312611	alpha-amylase like

2. No of sequences in metabolizing enzymes of starch and sucrose metabolism pathway.

Enzyme	No of seqs in enzyme	Stage of Growth
EC:5.3.1.9 - isomerase	9	young to mature stage
EC:2.4.1.34 - synthase	4	young to mature stage
EC:2.4.1.18 - branching enzyme	52	young to mature stage
EC:2.7.7.27 - adenylyltransferase	15	young to mature stage
EC:2.7.1.1 - hexokinase type IV glucokinase	5	young to mature stage
EC:2.7.1.4 - fructokinase (phosphorylating)	1	young to mature stage
Enzyme	No of seqs in enzyme	Stage of Growth
EC:2.4.1.34 - synthase	1	young to ripening stage
EC:2.7.1.1 - hexokinase type IV glucokinase	4	young to ripening stage
EC:2.4.1.12 - synthase (UDP-forming)	1	young to ripening stage
EC:2.4.1.25 - disproportioning enzyme	2	young to ripening stage
Enzyme	No of seqs in enzyme	Stage of Growth
EC:5.3.1.9 - isomerase	9	mature to ripening stage
EC:2.4.1.34 - synthase	4	mature to ripening stage
EC:2.7.7.27 - adenylyltransferase	15	mature to ripening stage
EC:2.7.1.1 - hexokinase type IV glucokinase	5	mature to ripening stage
EC:2.7.1.4 - fructokinase (phosphorylating)	1	mature to ripening stage
EC:3.1.3.24 - phosphatase	2	mature to ripening stage
EC:3.2.1.21 - gentiobiase	2	mature to ripening stage

EC:3.2.1.1 - glycogenase,	1	mature to ripening stage
EC:3.2.1.4 - endo-1,4-beta-D-glucanase,	2	mature to ripening stage
EC:2.4.1.13 - synthase	5	mature to ripening stage
EC:3.1.3.12 - trehalose 6-phosphatase,	1	mature to ripening stage
EC:3.2.1.26 - invertase,	1	mature to ripening stage
EC:3.2.1.2 - saccharogen amylase,	3	mature to ripening stage
EC:3.2.1.39 - endo-1,3-beta-D-glucosidase	2	mature to ripening stage
EC:3.2.1.48 - alpha-glucosidase	1	mature to ripening stage
EC:2.4.1.14 - synthase	4	mature to ripening stage
EC:3.2.1.20 - maltase	1	mature to ripening stage
EC:2.4.1.12 - synthase (UDP-forming)	14	mature to ripening stage
EC:2.4.1.1 - phosphorylase	4	mature to ripening stage
